# Simulating the Mammalian Blastocyst - Molecular and Mechanical Interactions Pattern the Embryo

**DOI:** 10.1371/journal.pcbi.1001128

**Published:** 2011-05-05

**Authors:** Pawel Krupinski, Vijay Chickarmane, Carsten Peterson

**Affiliations:** 1 Computational Biology & Biological Physics, Department of Astronomy and Theoretical Physics, Lund University, Lund, Sweden; 2 Division of Biology, California Institute of Technology, Pasadena, California, United States of America; 3 Lund Strategic Research Center for Stem Cell Biology and Cell Therapy, Lund University, Lund, Sweden; Ecole Normale Supérieure, France

## Abstract

Mammalian embryogenesis is a dynamic process involving gene expression and mechanical forces between proliferating cells. The exact nature of these interactions, which determine the lineage patterning of the trophectoderm and endoderm tissues occurring in a highly regulated manner at precise periods during the embryonic development, is an area of debate. We have developed a computational modeling framework for studying this process, by which the combined effects of mechanical and genetic interactions are analyzed within the context of proliferating cells. At a purely mechanical level, we demonstrate that the perpendicular alignment of the animal-vegetal (a-v) and embryonic-abembryonic (eb-ab) axes is a result of minimizing the total elastic conformational energy of the entire collection of cells, which are constrained by the zona pellucida. The coupling of gene expression with the mechanics of cell movement is important for formation of both the trophectoderm and the endoderm. In studying the formation of the trophectoderm, we contrast and compare quantitatively two hypotheses: (1) The position determines gene expression, and (2) the gene expression determines the position. Our model, which couples gene expression with mechanics, suggests that differential adhesion between different cell types is a critical determinant in the robust endoderm formation. In addition to differential adhesion, two different testable hypotheses emerge when considering endoderm formation: (1) A directional force acts on certain cells and moves them into forming the endoderm layer, which separates the blastocoel and the cells of the inner cell mass (ICM). In this case the blastocoel simply acts as a static boundary. (2) The blastocoel dynamically applies pressure upon the cells in contact with it, such that cell segregation in the presence of differential adhesion leads to the endoderm formation. To our knowledge, this is the first attempt to combine cell-based spatial mechanical simulations with genetic networks to explain mammalian embryogenesis. Such a framework provides the means to test hypotheses in a controlled *in silico* environment.

## Author Summary

We elucidate by computational means the processes by which the development of the mammalian embryo during its first four to five days occurs, as it is transformed from a single stem cell into hundreds of cells of different tissue types. We are interested in understanding the fundamental processes of how gene expression dynamics within each cell is coupled to the mechanical forces between cells, such that cells move to take up their positions as part of different tissues depending on the genes they express. Recent experiments which track single cell movement and division in conjunction with their gene expression dynamics suggest various hypotheses as to how this coupling functions to pattern the embryo. We have developed a computational model which can test these hypotheses. The model consists of dividing cells, interacting with each other through mechanical forces, within a confinement of embryo boundary. Each cell contains a genetic network of specific genes which influence cell adhesion properties and cell division plane directions. We explicitly simulate the formation of the trophectoderm and endoderm layers of cells which illuminates the principles by which the embryo is robustly patterned.

## Introduction

How a complete embryo emerges starting from a single fertilized egg is an intriguing process in developmental biology, understanding of which has important clinical implications [1]. Recent advances in live imaging have allowed for the tracking of single cells as they grow and divide and subsequently form different tissues of the embryo [2]. Using fluorescent labeling one is able to monitor in real time the expression levels of key transcription factors in single cells as they move and divide. Recent experiments have shown significant correlations between the individual cell fates and specific gene expression patterns [3], [4]. Studies with respect to early events in the morphogenesis of the mammalian embryo suggest that, although the combined interplay between gene expression and cell polarity perhaps determine the cell division rules, the mechanical properties of cells which may also depend on gene expression, collectively organize cells into different tissues [3]–[5].

**Figure 1:**
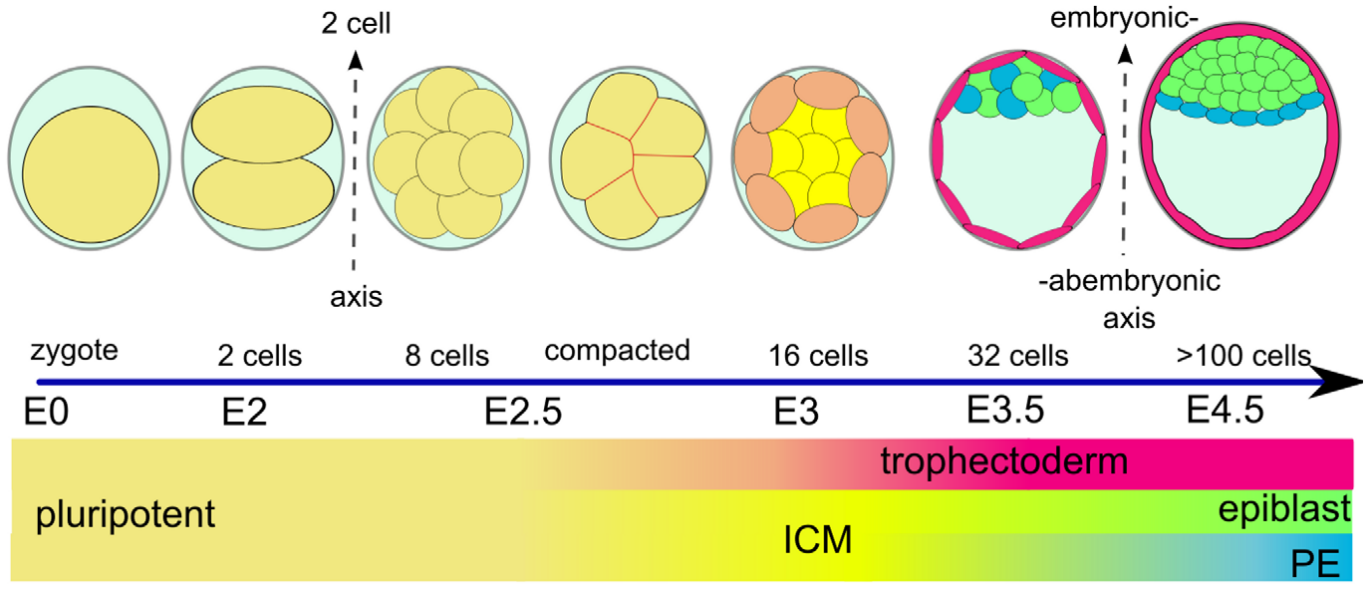
Schematic view of morphological and lineage specification steps during the early mouse embryonic development. Starting from fertilization (E0), after three rounds of cleavages (E1–E2.5), the blastomers undergo compaction and polarization (E3). Then the trophectoderm outer layer starts to separate from the inner cell mass (ICM) followed by the expansion of the blastocoel and the localization of the ICM to one part of the embryo (E3.5). After this stage the endoderm is formed as a layer separating epiblast from blastocoel (E4.5). After 4.5 embryonic days, the preimplantation embryo contains more than 100 cells.

The first developmental phase occurs when some of the cells from the morula differentiate to become part of the trophectoderm (TE) lineage, forming an outer layer surrounding the inner cell mass (ICM) [6] (Figure 1). After the TE layer is formed, cells secrete a fluid, which coalesces and expands as a single entity, the blastocoel [7]. The latter gradually pushes all ICM cells to one end of the protective outer envelope, the zona pellucida (Figure 1). At this stage a second developmental event occurs – the formation of the primitive endoderm (PE). This is the covering which separates the ICM from the blastocoel. The analysis of molecular and mechanical processes, which ensure the robust patterning of these layers of cells [3], is the subject of this work.

Previous studies have identified specific gene expression with the three lineages, ICM, TE and PE. The inner cells which ultimately give rise to the three germ layers are pluripotent and express the well known embryonic stem cell transcription factors *Oct4*, *Sox2* and *Nanog* amongst several others [8]. The cells forming the trophectoderm exclusively express *Cdx2*, whereas, the cells which are part of the endoderm lineage express *Gata6*. There are several mutually antagonistic interactions between these key transcription factors. *Cdx2* represses *Oct4* and vice versa [9]. In addition, these transcription factors are also positively auto-regulating (see [10], [11] and references therein), ensuring that once turned on, they remain stably expressed.

Recent work suggests that stochasticity is instrumental in the patterning process, in which key genes are initially expressed in a fluctuating manner and only later in the development does a pattern emerge [12]. When cells have decided upon particular lineages, the positive auto-regulation ensures that only the trophectoderm cells, which are the outer cells express *Cdx2* (simultaneously suppressing *Oct4*), whereas the inner cells express *Oct4* (suppressing *Cdx2*) thereby enforcing mutually exclusive expression of lineage genes [13]. Similarly, *Gata6* and *Nanog* are very likely mutually antagonistic [14], the former is expressed in PE whereas *Nanog*, which is part of the trio of embryonic transcription factors, is expressed in the epiblast cells. In [11] a computational model, based upon several interactions of these key genes, was developed for the genetic circuit which determines cell fate, i.e., TE, epiblast or PE. The main conclusion from [11] was that the network dynamics exhibited a switch-like behavior, as a function of an external signal. The question of interest in this work is how the circuit dynamics of these various components regulate cell fate, as cells become part of the PE, ICM and TE.

After the fertilized egg has undergone three rounds of division (Figure 1), the outer cells get polarized along the apical-basal direction. If an outer cell undergoes a *symmetrical* division, both daughter cells retain the cell polarity, but if the division is *asymmetrical*, the inner cell looses polarity. Cell polarity and *Cdx2* expression have been implicated to feedback onto each other [15], thereby making the polarized cells increase *Cdx2* expression. This ensures that the outer cells express *Cdx2*. However, this begs the question as to which factor determines *symmetrical/asymmetrical* divisions. In [15], the authors suggest that CDX2 levels themselves affect the division pattern. Cells, which express higher levels of CDX2, divide *symmetrically*, whereas for lower levels of CDX2, cells divide *asymmetrically*, such that upon division, the inner cell gives up most of its *Cdx2* mRNA to the outer cell. Although the mechanism by which cells choose their division plane by reading out the levels of CDX2 is not known, it can be classified as implementing the “cell lineage determines cell position” rule. An alternative is the “cell position determines cell lineage” rule, which is thought to be connected to nuclear localization of YAP, which is a cofactor of Tead, a transcription factor upstream of *Cdx2*. In [16], the authors suggest that cells that are outside lack a signal, which necessarily allows YAP to be localized to the nucleus. In this way, the outside cells automatically express *Cdx2*, whereas the inner cells do not express *Cdx2*. In both of the above hypotheses, it can be assumed that the *Oct4*- *Cdx2* mutual antagonism gradually fine tunes any small discrepancies in their levels, once they are determined to be expressed in cells.

The next stage of development is the PE formation. The transcription factor *Gata6* is expressed by the PE cells, which ensures through the mutual repressive interactions with *Nanog*, that the embryonic genes are shut off. However, initially, *Gata6* and *Nanog* seem to be expressed in spatial “salt and pepper” pattern [12]. From this initial distribution the pattern changes such that, the cells occupying the outer layer of the ICM, which face the blastocoel, must express *Gata6*. How cells get patterned in this manner, has been the subject of ample research [3]. Three different processes are thought to occur [17]. If the cells, which express *Gata6* have slightly different adhesive properties from the cells expressing *Nanog*, the two populations of cells can get sorted out. However, for some of the *Gata6* cells, which must move out from deeper layers of the ICM to occupy the outer layer, there could be some type of external “homing” signal. It is interesting to speculate if the fibroblast growth factor FGF signaling, which plays role in the endoderm development [3], might provide such a cue. Finally, cells expressing *Gata6*, which are unable to move through the deeper layers and emerge to the outer layer, can undergo apoptosis, and be hence removed from the entire population of cells. These processes can be combined to give a robust “movement” of GATA6 cells [18], thereby implementing the “cell lineage determines cell position” rule.

One of the aspects of embryo development is the formation of the embryonic-abembryonic (eb-ab) axis. An early proposal was that the eb-ab axis position was correlated to the first division plane of the fertilized egg [3]. Each of two cells would then contribute to different tissues (TE and epiblast) of the embryo. However, significant cell movement of individual cells occurs and the entire mass of cells can also rotate [2]. This makes it difficult to follow and assess the clonal expansion of cells. Another hypothesis is that the emergence of the eb-ab axis is entirely due to mechanical constraints [19]. The pellucid zone (ZP) is usually elliptical, and hence it is possible that cells move into one end of the long axis to minimize the elastic energy.

We propose a mathematical framework which takes into account growing and proliferating cells, interacting through physical forces to understand the patterning of the blastocyst into the trophectoderm, epiblast and the primitive endoderm. Two hypotheses which we explicitly explore are, (1) gene expression determines the geometry of division (2) gene expression determines cellular motion through modification of cellular adhesion properties. The gene expression itself is determined by an underlying genetic network, which is coupled to both division and spatial location within the embryo.

Faced with the complexity of the processes described above, we believe that such a computational approach, in which each hypothesis is simulated explicitly, provides the means to bridge intuition with understanding. Further, it provides novel predictions which can subsequently be tested.

## Model

The cell based model presented here aims at describing morphogenesis of the mammalian embryo in the early phases of development in terms of simplified mechanical interactions between blastomeres, which are coupled to gene network dynamics within each cell. The network dynamics feeds back on (i) division patterns and (ii) adhesive properties of cells.

The mechanical part of our model is inspired by Dallon and Othmer [20], who analyzed cell movement in the *Dictyostelium discoideum* slug. We model cells as elastic spheres interacting with each other and constrained by the pellucid zone. Spherical geometry faithfully reproduces the shape of the cells in early mammalian embryogenesis except for the flattened trophectoderm cells where the oblate elliptical shape is more appropriate. The blastomeres are treated as incompressible elastic bodies, whose mechanical response is confined to three orthogonal axes. This is equivalent to cells being represented as membranes whose deformation is restricted by springs in three orthogonal directions. These directions correspond to the principal directions of the stress tensor and all the external forces acting upon the cell are resolved to these coordinates. Cells come into contact and mechanically interact with each other (Figure S1). Keeping track of the attachment points of the forces on the surface of the cell allows the model to follow the changes in translative, compressive and tensile forces. The forces acting on the cell are summed up over all the neighbors of the cell. The neighborhood relation itself is determined from the Voronoi diagram of the cell centers, which is dynamically updated at each step of the simulation. Assuming that due to the low Reynolds number of the cell movement we can neglect accelerations in the dynamics we write the equations of motion for individual cells 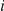 in the form,
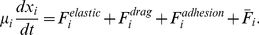
(1)


In Eq. (1) we account for three types of mechanical forces: elastic interaction between cells (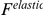), adhesive drag force of cells sliding against each other (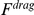) and an attractive adhesion force (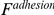). The 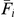 represents forces due to the active movement of cells or pressure from blastocoel, which do not originate from intrinsic mechanical interactions between the cells. The factor 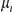 stands for the viscosity coefficient and 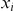 is the three-dimensional position vector of cell 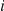 (see Text S1 for details).

Each blastomere is defined by the genetic network (see Text S1 for details), which evolves the mRNA and protein concentrations of the cell, a set of parameters (Table S1) which define its mechanical properties, a polarization vector and the cell cycle length. These parameters can be different for different cell types (i.e. TE, PE and ICM).

Cell division in our model (see Text S1 for details) is a discrete event where a single mother cell is replaced by two daughter cells. The cells undergo division when the time elapsed from the last division exceeds the cell cycle length. The latter is randomized among the blastomeres according to a normal distribution [21]. The total cell volume is conserved during this step and initially overlapping daughter cells occupy the space inside the mother cell. During division, in addition to the obvious change of geometry, cells also need to partition their content to the daughter cells. This is accomplished in the model using two different recipes: (i) *random symmetrical*, where the direction of division is random and the content is distributed symmetrically, and (ii) *polarized asymmetrical division*, where the direction of division is correlated with the cell polarization vector and the content is partitioned asymmetrically. Note that the division gives rise to opposing forces for both daughter cells, in a direction perpendicular to the division plane.

The mechanical equations of motion as well as genetic networks equations for the entire system are solved numerically using a fifth order Runge-Kuta differential equation solver, which makes adaptive time steps based on the requirement of keeping the error less than a given threshold. A typical movie of the resulting dynamics can be found in Video S1.

## Results

Our results discuss how the two important developmental stages of the embryo, namely the formation of the trophectoderm and endoderm, are dependent on the processes of cell division, gene expression and the mechanical interactions between cells within a confined space. We begin by considering purely mechanical interactions between cells, and how cell divisions affect their positioning within the embryo. Later we use this as a substrate upon which we add gene expression, by including a genetic network within each cell and further by allowing cells to interact with the external environment.

### Embryonic axes align due to the mechanical interactions

We first analyze the correlation between the orientation of the blastocyst embryonic-abembryonic (eb-ab) axis and the axis of the two-cell embryo. Our motivation is to test the hypothesis that this phenomena occurs from the alignment of both of these directions with the long axis of elliptical pellucid zone (ZP) due to the mechanical constraints. This hypothesis has been analyzed experimentally in several studies providing data both in favor and against it [17], [22]–[24]. The inconsistent results could be due to different strains of specimen, different experimental techniques, or difficulty in tracking cells given the considerable cell mobility in the early embryo and the embryo inside the ZP as a whole. However, this discrepancy could also be a result of different mechanisms that are involved in the formation of the eb-ab axes and the cleavage pattern of the two cell embryo. Since the shape of the ZP is not perfectly spherical, it provides a directionality, which could influence the orientation of the cells in the developing embryo. Here we test whether the mechanical constraints arising from the ZP geometry could be the underlying cause of the orientation of the embryo, both at two-cell and blastocyst stages.

**Figure 2:**
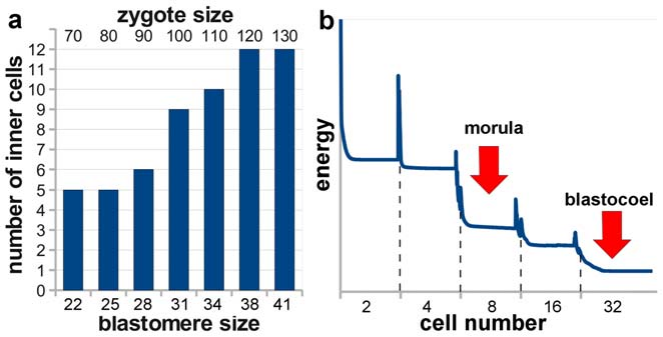
Examples of geometrical and mechanical effects in embryogenesis. (**a**) The number of inner cells in the simulations at the 32-cell stage (E3.5 in Figure 1) as a function of the blastomeres sizes. As the diameter of the cells increases, geometrical constraints force more cells to position themselves inside the cluster. (**b**) Average elastic deformation energy of the blastomeres (energy of the springs simulating elastic response of the cells) as a function of time in a typical simulation. The sharp peaks in energy correspond to cell division events. We observe a decline of the deformation energy perceived by blastomeres as the number of cells increase. Particularly large drop in energy takes place at 4- to 8-cell transition. The 32-cell stage shows the lowest deformation energy in this picture. Interestingly, these are the stages of development when morula compaction and blastocoel formation happen.

In [25] the authors developed a computational model of the embryo comprised of cells with a blastocoel, surrounded by the ZP to study mechanical effects on the eb-ab axis. They concluded that the cells would acquire the configuration of minimal energy orienting themselves along the corner of the ZP long axis. Hence, mechanical interactions would determine the eb-ab axis. Although their model demonstrates this interesting result, it does not take into account cell proliferation. Within our model, we are able to analyze the joint effects from mechanical interactions on orientation of both two cell embryo and the eb-ab axes together with cell proliferation. In our model, the ZP acts as a static barrier elastically repelling the spherical blastomeres in contact, while the cells interact mechanically with each other and favor configurations which minimize the elastic energy of the system (Figure 2b). Based on the fact that isolated blastomeres attain spherical shapes, the elastic blastomere energy in the model increases with the overlap of the spheres representing their native shape. Therefore the two first blastomeres will position themselves along the long axis of ellipsoidal pellucid zone minimizing their overlap or deformation. In the model we assumed that there are no frictional forces between ZP and blastomers, since our analysis of the motion of the cells in experiments and test simulations including friction suggest that such interaction has a marginal effect (see Text S1 for details). In such case, even tiny differences (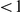
%) in the axes length of the ellipsoid were sufficient to provide a positional cue for the two cell embryo. As expected, due to the increase in blastomere overlaps, the dynamics of the alignment process is faster and more robust with increasing difference of axes length. The random division of outer cells, which are close to the ZP, can create a torque, which rotates the entire cell mass (Video S2). This rotation is also observed in experimental movies [2], which reaffirms that the mechanics within a confined region plays an important role in the arrangement of cells within the blastula.

**Figure 3:**
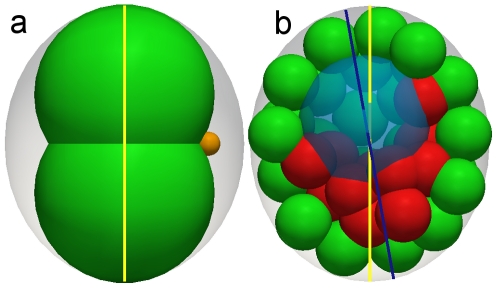
Alignment of the two-cell embryo symmetry axis and embryonic-abembryonic axis with the long axis of ellipsoidal pellucid zone. In the case of the two-cell embryo constrained by the elliptical pellucid zone (**a**) the positioning of the cells is consistently on opposite sides of the longest axis of ellipsoid by purely mechanical interactions and independent of the direction of the first division. The second polar body (a small cell with little genetic content positioned on the side of blastomers) is a product of the last meiotic division of the oocyte and marks polarization of the maternal mRNA. The blastocoel, which is modeled as a slowly expanding sphere inside of the embryo (**b**), positions itself preferentially at one end of the same axis. The frequency of this effect depends upon the elongation ratio of the pellucid zone and parameters of the model. However, it is consistently above 50% suggesting that orientation of the embryonic-abembryonic axis is mechanically biased as well.

As the development of the embryo progresses, the interactions between blastomeres become more complex due to both their increased number and changes in their mechanical properties. Around the 32-cell stage, with the trophectoderm well defined, the fluid filled blastocoel cavity begins to form with secretion of intracellular vacuoles which coalesce. We model the blastocoel in a simplified manner, as a slowly expanding spherically shaped region inside the ICM, aiming to capture the behavior of spatially restricted ICM cells. The adhesion strengths of the cell-cell interactions are deduced by what is qualitatively known for different cell types [12], [26]. Compacted ICM cells exhibit strong self-adhesion, trophectoderm cells adhere to each other through tight junctions and have decreased adhesion to ICM cells. While we do not expect any adhesion-like force between the cells and the blastocoelic fluid. The simulations are initialized with a small spherical blastocoel volume in the center of the ZP at the 32-cell stage that later expands to (20%–30%) of the whole embryo. Depending upon the degree of ZP elongation, we observe preferential localization of the blastocoel to the one end of the ZP long axis. To ensure that our results do not depend upon the initial 32-cell configuration, for each simulation we used a different initial template obtained from a single blastomere by five rounds of cleavages with stochastic time and direction of the cell division (see Text S1 for details). A 10% difference in the length of axes of the ellipsoidal ZP provides alignment of ab-eb axis to the long axis of ellipsoid (Figure 3, Video S3) in 76% (n = 50) of the simulations, suggesting that this configuration is mechanically preferred and that the oblate shape of the ZP could influence alignment of both the two-cell embryo and the blastocyst axes. We consider two axes aligned, for the purpose of the simulation, if the angle between them is less than 10 degrees and we define embryonic-abembryonic axis as the line passing through blastocoel center and center of mass of ICM cells. One should mention that *in vivo*, the ZP is not essential for blastocyst formation [2]. In our model we can also form a blastocyst without the ZP. In that case we do not observe the alignment of its axis with the axis of two cell embryo (Video S4). We conclude that in cases where the ZP is present, its shape affects the relative position of ICM and blastocoel in agreement with findings from the model in [25]. The same mechanical constraint aligns axis of two cell embryo and, as cells with the same lineage tend to occupy nearby positions, it influences lineage allocation to trophectoderm and ICM. This we confirm by lineage tracking in our simulations.

### Mechanics and geometry play important roles in embryo development

In relation to the orientation of eb-ab axis and blastocyst linage formation, timing and orientation of two- to four-cell divisions has been studied by several groups [21], [27], [28]. In particular, certain patterns of cleavages, meridional-equatorial (M-E) together with reversed equatorial-meridional (E-M) order, were found to occur more often (80% of cases) and were associated with specific tetrahedral arrangement of blastomeres in the blastula. In our model we find that the configuration of blastomeres in four-cell embryos depends upon the size of the blastomeres relative to the ZP and upon the ZP shape even more than upon the division pattern. We characterize the size of four-cell embryo blastomeres with respect to the size of the initial spherical zygote, 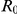. After two rounds of cleavages and conserving the cell volume we obtain blastomeres of the radius 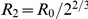. As a maximal radius of the zygote we consider the radius of the sphere of the same volume as the ZP. In simulations we used 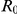 in range 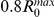 to 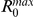 and varied the elongation ratio of the ellipsoidal ZP within 20%. In the case of spherical ZP, due to the symmetry, cells always attain tetrahedral configuration in four-cell embryo simulations. However, even a slight deviation (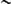
5%) from spherical ZP symmetry causes blastomeres to prefer different configurations minimizing the total elastic energy. By decreasing the blastomere sizes at this point, their mobility is increased since the drag force decreases and the elastic interaction between them is lowered. This is sufficient to rescue the tetrahedral arrangement at some point (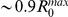). The exact numbers depend upon simulation parameters but the trends are robust. In the regime of large blastomeres, when they tend to depart from a tetrahedral configuration, their mobility is lowered and specific patterns of cleavages become increasingly important for their final configuration. Our results suggest that geometrical factors like size of the blastomeres and specific shapes of the ZP, ignored in studies of blastocyst lineage so far, may be influencing positions of the blastomeres in the blastula.

Similarly we analyzed the number of cells located inside and outside at the 32-cell stage as a function of the individual blastomere size. We found that the number of inner cells decreases when lowering the cell size (Figure 2a). While the spherical approximation may not accurately describe the flattened shape of the trophectoderm cells and we cannot expect to reproduce observed ratio of inner to outer cells in this way, the result again confirms that mechanical and geometrical constrains very likely play prominent roles in blastocyst development.

Early mammalian embryo formation is characterized by a sequential order of morphological events, such as morula compaction or blastocoel expansion, which take place at precise stages of development. Even if these events are under the genetic control of processes inside each blastomere, the exact mechanism governing them is unknown and it is possible that cues other than genetics can contribute to triggering those events. Our model offers the possibility to analyze mechanical interactions taking place during embryogenesis. We have measured the average energy per cell of elastic deformation of identical blastomeres, as a function of time, from 2- to 32-cell stage (Figure 2b). High peaks in this energy are observed during cell division, because just after a division, the daughter cells are highly deformed from their native spherical shape. More interestingly, we find differences in the stress perceived by blastomeres at different stages. We see an overall decrease of average deformation energy as the simulation progresses. This is expected since as blastomeres are getting smaller during cleavages, they can fit the pellucid zone shape with less overlap on average. In Figure 2b we observe larger differences in deformation energy between 4- to 8-cell and 16- to 32-cell stages than between 2- to 4-cell and 8- to 16-cell stages. Also note that the average cell deformation energy is lowest for the 32-cell stage. Since morula formation and compaction of blastomeres happen precisely at the 8-cell stage and the secretion of vacuoles forming blastocoel takes place approximately at the 32-cell stage, these results raise the intriguing question: Can sensing of mechanical signals provide triggers for some of the important events during embryogenesis?

### Is trophectoderm formation determined by cell position or cell division pattern?

**Figure 4:**
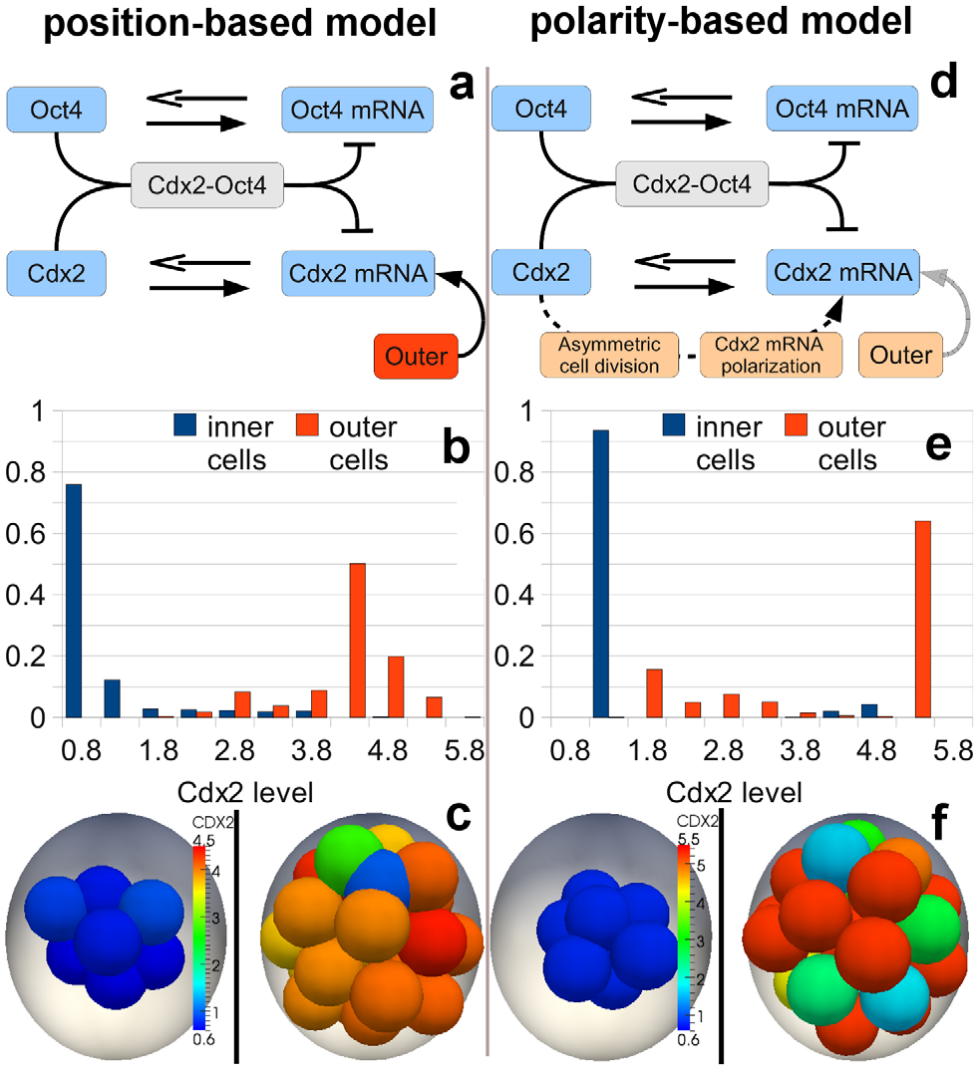
Separation of CDX2 distributions in inner and outer cells, with respect to embryo mass, in the position-based (a, b, c) and polarity-based models (d, e, f) respectively. The core of the simplified genetic network used in the simulations of the both models is based on mutual repression of *Cdx2* and *Oct4* and auto-regulation of both transcription factors. In the position-based model (**a**) outer cells receive an additional signal enhancing the expression of *Cdx2*. In the polarity-based model (**d**) this signal is reduced. Instead subcellularly polarized *Cdx2* mRNA levels can be affected during asymmetric division. (**b**) Normalized histogram of CDX2 levels in inner and outer cells from the position-based model. One observes a clear shift of distributions towards lower concentrations in inner cells and higher concentrations in outer cells. Both distributions are well separated. (**c**) Example of CDX2 expression in inner and outer cells at the 32-cell stage in the position-based model. Inner cells (left pane) typically have lower expression of *Cdx2* resulting from higher expression of *Oct4*. Outer cells (right pane) on average have higher CDX2 expression. (**e**) In the polarity-based model the distributions of CDX2 in inner and outer cells are statistically distinct, but show larger number of low CDX2 level cells in the outer position. (**f**) The polarity-based model results in higher CDX2 expression in outer cells (right pane) than in inner cells (left pane) on average, but typically also yields more outliers.

Trophectoderm is the first occurring specialized tissue distinguished from the embryo mass. Despite remarkable progress in our knowledge about this process, the exact mechanism of trophectoderm formation is still an area of active research. Here we evaluate the robustness of two conceptual models of *Cdx2* expression pattern during this process. In the first, “position-based model” the position of the cell in the embryo dictates the *Cdx2* expression level, with inner cells having lower expression levels of *Cdx2* than the outer cells [13]. We have implemented this model with a simple switch-like genetic network based on the mutual repression between *Cdx2* and *Oct4* in each cell (Figure S2, Figure S3, Text S1, Table S3) [8]. We assumed that the outer cells receive additional signal from polarity genes which enhances *Cdx2* expression in those cells (Figure 4a). Starting with small and random CDX2 levels at the 4-cell stage, we evolve the system to 32 cells. Since the relation between position and CDX2 level in the cell is defined in a straightforward manner in this model, we expect the distribution of CDX2 concentration to be clearly separated between the inner and outer cell populations. Indeed, we consistently observe (Figure 4b,c, Table S2) significantly higher levels of CDX2 at the 32-cell stage in outer cells, in agreement with experimental findings [13]. In the second, polarity-based model the spatial pattern of CDX2 is not determined by a direct relation to the position of the cell [15]. Instead, outer cells, which are known to be polarized, are assumed to polarize *Cdx2* mRNA as well and can distribute it non-uniformly between daughter cells during asymmetric divisions. To simulate this behavior, the daughter cell located outside receives 90% of the mother cell's *Cdx2* mRNA while the other daughter cell, placed during division inside the embryo mass, obtains the remaining small portion of original *Cdx2* mRNA content. In addition, the probability of symmetric division is small for low- and large for high- *Cdx2* expressing outer cells. Inner cells, due to the lack of polarization, always divide in a symmetric manner. Such a feedback loop between the CDX2 level and the inside-outside polarization has been proposed to produce high CDX2 content in outer cells. We tested this model taking into account cell movement and the physical constraints. In our simulations we adapted the gene network involving *Cdx2*, *Oct4* and the mRNA produced by these genes, as a bistable switch (Figure 4d, Text S1, Table S4). We tested whether the unequal distribution in the *Cdx2* mRNA levels in daughter cells after an outer cell divides, is sufficient to establish the observed pattern of *Cdx2* expression. As before, assuming random CDX2 levels at the 4 cell stage, we evolve the model up to the 32-cell stage and performed an analysis of the *Cdx2* expression level. The polarity-based model is, in principle, capable of creating distinct *Cdx2* expression levels of inner and outer cells (Figure 4e,f). However, since it results in larger overlap of *Cdx2* distributions in inner and outer cells, it is less robust than the position-based model, which by definition produces the required pattern. This behavior of the polarity-based model is a consequence of more indirect relationship between the CDX2 concentration and the cell position. This complicates conditions for trophectoderm specification, as it requires coordination of several factors, including mechanical interactions, which in addition to division patterns influence the final positions of the cells in the embryo. For example, to minimize relocation of low level CDX2 cells from inside to the outside and high level CDX2 cells in the opposite direction, due to the geometrical and mechanical constrains, the division patterns must take into account the ratio of the inside and outside cells. We explored the parameter space of the probability of symmetric and asymmetric cell divisions as a function of the CDX2 concentration within our model (Figure S5) and found that it had to be carefully tuned to obtain the optimal pattern. Another key point is that this model, in the form presented above, does not have a mechanism which could cope with high level CDX2 cells located inside, which indicates that it may need additional hypotheses in order to better match the observed CDX2 level distributions. We further show through a simplified population based model that this process is not as robust as the position-based model (Text S1, Figure S6). Finally, we should note that assumptions of both position- and polarity-based models could work in unison to produce the correct spatial pattern of CDX2 expression.

### Mechanical cues induce cell separation during endoderm pecification

The next major developmental event is the formation of the primitive endoderm (PE), which, from a patterning perspective, is very different from the trophectoderm patterning discussed above. Trophectoderm formation involves differentiating between inner cells and the outer cells that completely surround these. These cells take on very different fates. The endoderm is a layer of cells which separates the blastocoel from the ICM cells, and hence chooses a specific side, namely the one facing the blastocoel, thereby breaking the symmetry which is present in trophectoderm formation.

Two genes are characteristic in specifying the cell fate. *Nanog* which specifies the ICM cells and *Gata6* the cells which finally form the endoderm. Cells express these genes in a salt-and-pepper manner prior to the creation of the endodermic layer of cells next to blastocoel [8], [29]. Cells expressing *Gata6* are thought to migrate away from the *Nanog* expressing cells towards the blastocoel, through mechanisms of dynamic rearrangement to finally form the endoderm layer. There could be potentially several processes by which such a rearrangement is possible. We have used our mathematical framework to test each process individually as well as in combination so as to provide a comparison of different scenarios. The two mechanisms we have considered are differential adhesion, and active cell movement determined by a directional signal emanating from the blastocoel. Cell sorting with differential cell adhesion [30]–[32] has been shown to be an important mechanism to spatially separate two different cell types within heterogeneous populations. However, it is not obvious, how efficient such cell sorting is in a system the size of the ICM, where the motility of cells is considerably reduced due to the tight packing.

To test these hypothesis, we make the assumption that gene expression determines mechanical properties through differential adhesive properties of the Nanog and *Gata6* expressing cells. In addition, we test how the dynamics of interactions with blastocoel can affect the endoderm formation. This we do by making two alternative hypothesis. The first assumes that the blastocoel is a static surface which merely provides a barrier to moving cells. We also include a directional force on *Gata6* cells towards the blastocoel, by assuming that an external signal informs these cells to preferentially move in that direction. Although there is no evidence for such a directional signal, the role of growth factors could be instrumental in providing cues for directional cell movement. The second assumes that the blastocoel acts dynamically by exerting a constant and dynamic pressure on cells, thereby explicitly simulating forces between the ICM cells and the blastocoelic fluid.

**Figure 5:**
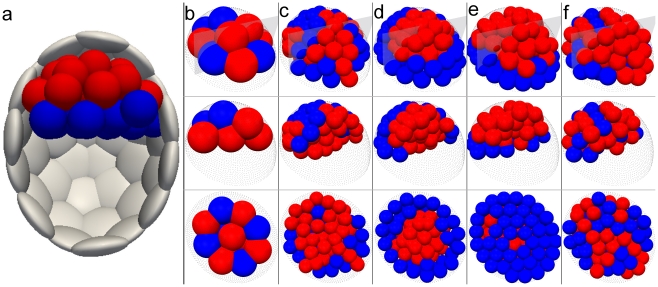
Effect of differential adhesion on separation of GATA6 (blue) and NANOG (red) cells during simulation with static blastocoel providing just a positional restriction for blastomers. (**a**) Schematic view of the late blastocyst. The differentiated trophectoderm (gray) and endoderm (blue) constrain the epiblast (red). (**b**) Initial configuration of 12 cells used in all presented simulations. The dotted shape shows the constraining surfaces of trophectoderm and blastocoel. In the middle pane, part of the cells were hidden to exhibit the interior of a cell cluster. The bottom pane shows the view from the blastocoel side. The NANOG and GATA6 cells are positioned in a salt-and-pepper pattern. The cells are allowed to take three rounds of division and move according to mechanical interactions within the constraining surroundings. The daughter cells are assumed to retain the identity (NANOG or GATA6) of their mother cells. In **c**, **d**, **e** and **f** the final state of the simulation is presented for different cases. (**c**) Adhesion coefficients are the same for both types of cells making them mechanically equivalent. Both NANOG and GATA6 cells are taking positions inside and outside in the cell cluster. (**d**) The NANOG cells have stronger self adhesion than GATA6 cells. The cross-adhesion between both cell types is small as compared to the average of the self adhesions. GATA6 cells form a layer of cells outside the NANOG cells next to the boundary. However, localization of this layer is not always close to the blastocoel boundary (middle and bottom panes). This suggest that differential adhesion and boundary constraints are enough to separate the two populations of cells, but are not sufficient to position GATA6 cells in the direct proximity of blastocoel. (**e**) Combination of differential adhesion and directional movement of GATA6 cells towards a signal from the blastocoel boundary is able to position all the GATA6 cells next to blastocoel boundary. (**f**) A directional signal alone is not sufficient to achieve the same effect.

**Figure 6:**
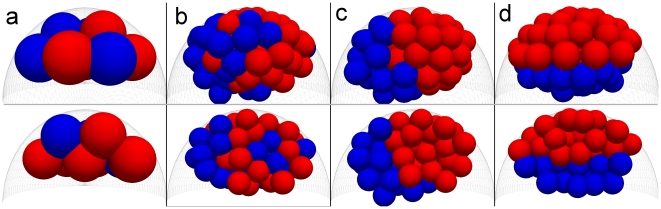
Results of simulation of endoderm formation in case of dynamic interactions of cells with blastocoel and random active movements of cells. The cells next to blastocoelic surface are pushed with forces proportional to their cross section surface area in direction perpendicular to the surface of the blastocoel. The bottom panes show the cross section through the cell mass to visualize the internal pattern. The GATA6 cells are blue and NANOG cells are red. (**a**) Initial state for the simulation. Both populations of cells are placed in the “salt and pepper” pattern. (**b**) Effect of random active movements alone. (**c**) Differential adhesion is able to form layer of GATA6 cells, but position of this layer is not stable with respect to blastocoel often resulting in ill placed endoderm. (**d**) Addition of stronger adhesion between NANOG cells and trophectoderm provides stabilizing signal which is able to place endoderm in correct position next to blastocoel.

In our model we consider an initial template of 10–14 cells constrained within a half-ellipsoidal space spanned by trophectoderm and blastocoel boundaries (Figures 5a and 5b). The ICM cells express either *Gata6* or *Nanog*, and are randomly distributed. Subsequently, cells move due to the processes described above and due to random motions which arise during cell divisions up to three division cycles, finally giving rise to 80–112 cells. During division the model assumes that the daughter cells retain the identity (*Nanog* or *Gata6*) of the parental cell. Throughout this process we assume the trophectoderm does not take an active part in the PE formation, since it is already specified prior to this stage. However, we include the interaction of blastomers with trophectoderm cells explicitly via elastic, adhesion and drag forces. Efficient cell sorting with differential adhesion requires a hierarchy of self and cross adhesion strengths [33]. We assume that cells expressing *Nanog* adhere strongly to each other, whereas cells expressing *Gata6* adhere less tightly to each other and the cross-adhesion between the two different cells types is the least. In the static blastocoel case, simulations of cell sorting suggest that (compare Figures 5c and 5d, Video S5) differential adhesion does have a positive role in segregating cells, although, by itself, does not position *Gata6* cells in endoderm layer. The patterning of the endoderm can be significantly improved on adding to differential adhesion a directional force attracting *Gata6* cells towards the blastocoel (as discussed above, Text S1), which gives the most robust result (Figure 5e, Video S6). Finally, the directional force alone (Figure 5f, Video S7), would not be sufficient, pointing to the fact that both, differential adhesion as well as the directional force are required to correctly position the *Gata6* cells adjacent to the static blastocoel barrier (Table 1). In fact the efficiency by which the endoderm layer is formed, can be increased considerably for a given directional force by tuning differential adhesion such that *Nanog* expressing cells adhere even more tightly while the adhesion between *Nanog* and *Gata6* cells is reduced(see Figure S7). Considering the second hypothesis, in which the blastocoel is a dynamic entity and exerts pressure on blastomers, we first test the effects of differential adhesion alone. The Figure 6c shows that although *Gata6* and *Nanog* expressing cells successfully segregate, the endoderm layer becomes tilted with respect to embryonic-abembryonic axis, such that *Nanog* expressing cells come into contact with blastocoel. Hence, the endoderm pattern is not successfully reproduced. If, however, we now assume a positional bias for *Nanog* cells such that they have stronger adhesion with the trophectoderm cells, we obtain the correctly patterned endoderm (Figure 6d). The mechanical forces on the ICM cells coming from the blastocoel push all the cells into one side of the embryonic-abembryonic axis. The *Gata6* cells, which neither adhere strongly to themselves, nor to the *Nanog* cells are pressed away from the strongly self-adhering and clustered *Nanog* cells, towards the blastocoel.

**Table 1:** The relative number of mispositioned cells (GATA6 cells in epiblast and NANOG cells in endoderm) at the end of simulation.

model	random	diff. adh	dir.mov.	diff. adh+dir.mov.
GATA6 in epiblast	42%	31%	21%	8%
NANOG in endoderm	51%	37%	27%	7%

The simulation starts from 12 to 14 ICM cells half of which are NANOG and another half GATA6. The cells are allowed to take three rounds of divisions and then their position (endoderm or epiblast) and gene expression (GATA6 or NANOG) is recorded. Number of mispositioned GATA6 (NANOG) cells relative to the total number of epiblast (endoderm) cells is presented for simulations of different separation mechanisms. Numbers for each model are averages of 6 simulations. In random model (first column) cells are labeled as GATA6 and NANOG but there is no difference in their adhesion strengths or directional movement. The differential adhesion model (second column) assume that GATA6 cells have slightly lower self-adhesion than NANOG cells. Cross-adhesion between both types is small as well. The directional movement model without differential adhesion (third column) features constant force drawing the GATA6 cells towards endoderm position. The lowest number of misplaced cells is obtained by combination of differential adhesion and directional signal (fourth column).

We also analyzed the effects of additional random forces, simulating cumulative effect of active cell movements and interactions with the environment. As can be seen from the Figure 6b, random forces by themselves do not suffice for the formation of the endoderm pattern, as they cannot provide the directional signal and are inefficient in allowing the *Nanog* cluster to form. The main effect of the random forces is to increase the efficiency of the pattern formation. This is because the extra random motions allow cells to sample more locations in space, leading to a better search for energetically favorable positions, thereby forming the endoderm much faster.

These results indicate that, even in the small sized system of ICM, differential adhesion is a crucial mechanism, which can sort ICM and endoderm cells. However, to do it efficiently and to position the endoderm in the correct place requires, in addition to differential adhesion ,either a directional force on *Gata6* cells, assuming that the blastocoel surface is a static barrier, or a dynamic interaction with the blastocoel and preferential adhesion of *Nanog* and TE cells. Comparison of these two different hypotheses, suggests that robust endoderm formation can occur without the presence of a directional chemical signal, purely though mechanical interactions arising from differential adhesion, but only if we include the dynamics of the entire embryo.

Finally, although we have not explicitly modeled apoptosis, we expect that it would play a synergistic role in the endoderm layer formation along with differential adhesion and the active motion. Its function could be to eliminate cells that are placed in the wrong position and hence cannot move towards their target destination, due to the forces arising from intervening cells. It could also increase motility of cells by freeing up space, or ensuring the correct ratio of ICM to PE cells during endoderm formation.

## Discussion

The emergence of high quality experimental data of developing embryos is an opportunity to develop mathematical models which can be used to elucidate the different mechanisms of embryogenesis and their interplay. Our model describes how gene expression, cell proliferation and mechanical cell properties contrive to provide structure and patterns to the embryo as it morphs from a single cell to pattern of two tissue types - the trophectoderm and the endoderm.

We first discussed how the observed correlation between the directions of the two cell embryo (animal-vegetal) and embryonic-abembryonic axes is explained as a mechanical effect of cell mass alignment with the long axis of the ellipsoidal pellucid zone. Cells find their position as they continuously move to decrease the mechanical stress that arises when cells are pushed against each other in a constrained space. Hence mechanical constraints are important in patterning the embryo. While this was shown in connection to blastocoel positioning, in [25], we have here advanced the understanding by including cell proliferation, explicitly taking into account cell divisions. An advantage of this is that one can address alignment of the embryo continuously at all the stages of development and perform lineage tracking of individual blastomeres. This allows for more detailed comparisons with live imaging experiments.

Next we considered the formation of the trophectoderm through two different mechanisms. The first is the position-based hypothesis, which asserts that cells on the boundary of the inner cell mass express *Cdx2*, which commits these cells to form the trophectoderm. Hence the position of a cell determines its fate. We also considered the second hypothesis, the polarity based model, where cell division directions are regulated by CDX2 levels, such that cells on the outside of the inner cell mass having high CDX2 levels divide symmetrically, thereby positioning both daughter cells with high CDX2 levels on the outside. For cells with low CDX2, division occurs asymmetrically, such that one cell is on the outside and inherits more *Cdx2* mRNA, whereas the inner cell has less *Cdx2* mRNA. In this way gene expression determines the fate of the cell, and ultimately its position within the embryo. Although both models produce enhanced CDX2 levels in outer cells, the position-based model is more robust. This is due to the deficiency of the polarity-based model in dealing with high CDX2 cells in the ICM. Consider in the polarity-based model an inner cell, which due to stochastic fluctuations expresses high CDX2. Then this cell would divide symmetrically, thereby ultimately supplying inner cells with high CDX2. Our simulations validate this hypothesis, suggesting that the polarity-based alternative, by itself, is less plausible as the mechanism by which cells find themselves as part of the trophectoderm lineage (Figure 4, Figure S4, Figure S5, see also discussion in Text S1). Our model suggests that experiments in which either *Cdx2* is transiently upregulated in inner cells or *Cdx2* is transiently downregulated in outer cells, and the cell division patterns followed by live imaging, would test the hypothesis of this model. Further, *in silico* the ratio of inner and outer cells could be simulated, thereby elucidating the control of division patterns by CDX2 concentration.

Finally we modeled the formation of the endoderm through processes which couple gene expression with the motility of cells. We first considered differential adhesion between cells which express *Gata6*, ultimately forming the endoderm layer, and *Nanog*, which form the ICM. From our simulations, which included a range of adhesion strengths, we inferred that with strong NANOG-NANOG adhesion, weaker GATA6-GATA6 adhesion and still weaker cross adhesion, the two cell populations segregate. We found, however, that in the case of the static blastocoel, the robust formation of endoderm layer required some sort of directional force on *Gata6* expressing cells, which could be postulated to arise from a hypothetical signal from the blastocoel. This we implemented through an extra force which moves *Gata6* cells towards the blastocoel. We also found that another robust way of a directional bias guiding *Gata6* cells towards the blastocoel could be obtained through purely mechanical means. Here we introduced a new model, in which cells in the ICM dynamically pushed in a direction away from the blastocoelic surface. We also found here, that active random motions of the cells allowed the endoderm pattern to be formed more quickly and robustly. Designing an experiment to test which of these two mechanisms does the embryo employ in actually forming the pattern is an interesting question for the future. We should point out that although we do not include apoptosis in our model, we expect that its inclusion would result in the elimination of remaining outlying cells which do not reach the endoderm and could facilitate movement by freeing up space for other cells.

Mathematical modeling offers an unique opportunity to test and compare experimentally based hypotheses in a controlled *in silico* environment. As more detailed data becomes available accurate and predictive models of embryonic development would enhance our understanding of early mammalian embryogenesis. This work is based on different modeling techniques that have been used separately in other contexts, but it is to our knowledge, the first attempt to combine cell-based spatial mechanical simulation with a genetic network approach within the same computational framework to explain mammalian embryogenesis. Within this single framework, we are able to integrate seamlessly several very different temporally separated developmental events (Video S8). This enabled us to address not only intermediate stages but also the final pattern, as various genetic processes unfold in time.

Our model for the formation of different tissue types can be advanced in several directions. We have assumed so far that the ZP is fixed in its geometry. This simplification has allowed us to avoid tracking changes in the shape of the ZP due to the forces between the latter and the cells inside. We aim to implement this feature in the future, which would be important for the later stages of development such as the transition from the blastocyst to the early egg cylinder [3]. In silico tests, such as the application of external forces on the ZP, and the effect on movement of cells and consequently formation of the endoderm layer, would be useful in comparison with experiments. The other challenging problem would be to simulate the changing morphology of trophectoderm cells as they morph from roughly spherical to slightly flattened out cells. The effects of their shape and also tight junctions between them could be an important factor in analyzing in the model. One future goal we plan to pursue is to model the formation and movement of the visceral endoderm [3]. Since our framework allows the implementation of signaling and gene expression networks within cells, it would be interesting to include signaling due to Nodal, bone morphogenic protein (BMP) and WNT and their roles in patterning the ICM. As pointed out by Cockburn et al [1], the formation of the three lineages is crucial for the development of the fetus. We hope that our *in silico* approach of studying the dynamics of cells due to different hypothesis could ultimately prove useful in a clinical setting.

## Supporting Information

Figure S1Schematic representation of the geometrical constructs used in force calculation of two interacting ellipsoidal cells. The geometry of each two cell intersection is approximately described in terms of vector between their centers 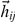, vectors between each cell center and a point on the ellipsoid closest to other ellipsoid's center 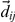, 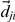, and the vector between surfaces of the cells 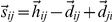. The two cells are intersecting if 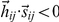.(0.01 MB PDF)

Figure S2Bifurcation diagrams of both protein and mRNA equilibrium levels as a function of polarity signal P. Moderate values of P (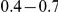) result in bistable behavior of the system.(0.01 MB PDF)

Figure S3 Time evolution of expression levels of *Cdx2* and *Oct4* showing switching from low to high CDX2 state and vice versa in position-based model network, (a) and (b), as well as in polarity based model (c) and (d).
(0.04 MB PDF)

Figure S4 Histograms of CDX2 levels in the simulations of position-based (a) and polarity-based (b) models for the fast genetic network dynamics. Compare with Fig. 4 in the main text showing the case of the slow network.
(0.04 MB PDF)

Figure S5 Aberrations of CDX2 levels in trophectoderm formation simulations in the case of non-optimized parameters in the polarity-based model.
(0.02 MB PDF)

Figure S6Results of the Monte Carlo analysis of the ratio of inner to outer cells at the 32-cell stage of embryo development as a function of the probability of asymmetric division. The theoretically estimated ratio of 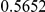 is marked by red horizontal dashed line. It crosses both the theoretical solution for corresponding asymmetric division probability (blue solid line) and the mean value of Monte Carlo simulations (pink stars) at 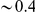. The pink solid lines mark the standard deviation of the Monte Carlo results. See Text S1 for details.(0.01 MB PDF)

Table S1 The ranges of the mechanical parameters used in the simulations.
(0.03 MB PDF)

Table S2 Statistics of CDX2 expression in simulations of trophectoderm specification in cell polarity-based (gray columns) and position-based (white columns) models from 100 simulations.
(0.02 MB PDF)

Table S3 Parameters used in the simulations of the gene network in the position-based trophectoderm formation model. The parameters in the top table are the same as in polarity-based model.
(0.03 MB PDF)

Table S4 Parameters used in the simulations of the gene network in the polarity-based trophectoderm formation model.
(0.03 MB PDF)

Text S1 Supporting text.
(0.14 MB PDF)

Video S1 Simulation of embryonic development up to 32 cell stage. First pane from left shows CDX2 levels in cells. Second pane shows inner and outer cell status as well as polarization directions. Third pane displays nuclei with different colors representing lineage of four cell embryo.
(4.05 MB AVI)

Video S2 Simulation of embryonic development prior to blastocoel formation. Notice rotation of the cell mass at each stage resulting from interaction with pellucid zone.
(1.75 MB AVI)

Video S3 Simulation of blastocoel emergence in the presence of the pellucid zone. Alignment of two cell embryo and embryonic-abembryonic axes with long axis of the pellucid zone is apparent.
(5.79 MB AVI)

Video S4 Simulation of blastocoel development without pellucid zone. In this case embryonic-abembryonic axis becomes almost perpendicular to two cell embryo axis.
(6.99 MB AVI)

Video S5 Simulation of endoderm formation by differential adhesion alone. GATA6 (blue) cells have lower self adhesion than NANOG (red) cells and cross adhesion between both cell types is lowest of all. GATA6 cells separate from the cell mass to the outside of available space, but are not all placed next to the blastocoel surface (bottom) as required for correct endoderm specification.
(3.84 MB AVI)

Video S6 Simulation of endoderm formation in the presence of both differential adhesion and directional signal towards blastocoel. The parameters used in the simulation are the same as in two previous movies. All the GATA6 cells are robustly moved to the blastocoel surface.
(3.86 MB AVI)

Video S7 Simulation of endoderm formation with directional signal towards blastocoel only. Puling of the GATA6 cells downwards is not sufficient in this case to move all the cells next to blastocoel boundary.
(3.30 MB AVI)

Video S8 An example of a complete simulation of embryo development from 1 to 128 cells. Simulation includes trophectoderm formation in “position-based” model, blastocoel growth and endoderm formation by differential adhesion and directional signal mechanisms.
(8.92 MB AVI)

## References

[1] Cockburn K, Rossant J (2010). Making the blastocyst: lessons from the mouse.. J Clin Invest.

[2] Kurotaki Y, Hatta K, Nakao K, Nabeshima Y, Fujimori T (2007). Blastocyst axis is specified independently of early cell lineage but aligns with the ZP shape.. Science.

[3] Rossant J, Tam PP (2009). Blastocyst lineage formation, early embryonic asymmetries and axis patterning in the mouse.. Development.

[4] Zernicka-Goetz M, Morris SA, Bruce AW (2009). Making a firm decision: multifaceted regulation of cell fate in the early mouse embryo.. Nat Rev Genet.

[5] Yamanaka Y, Ralston A, Stephenson RO, Rossant J (2006). Cell and molecular regulation of the mouse blastocyst.. Dev Dyn.

[6] Marikawa Y, Alarcón VB (2009). Establishment of trophectoderm and inner cell mass lineages in the mouse embryo.. Mol Reprod Dev.

[7] Motosugi N, Bauer T, Polanski Z, Solter D, Hiiragi T (2005). Polarity of the mouse embryo is established at blastocyst and is not prepatterned.. Genes Dev.

[8] Niwa H (2007). How is pluripotency determined and maintained?. Development.

[9] Niwa H, Toyooka Y, Shimosato D, Strumpf D, Takahashi K (2005). Interaction between Oct3/4 and *Cdx2* determines trophectoderm differentiation.. Cell.

[10] Chickarmane V, Troein C, Nuber U, Sauro HM, Peterson C (2006). Transcriptional dynamics of the embryonic stem cell switch.. PLoS Comput Biol.

[11] Chickarmane V, Peterson C (2008). A computational model for understanding stem cell, trophectoderm and endoderm lineage determination.. PLoS One.

[12] Dietrich JE, Hiiragi T (2007). Stochastic patterning in the mouse pre-implantation embryo.. Development.

[13] Ralston A, Rossant J (2008). *Cdx2* acts downstream of cell polarization to cell-autonomously promote trophectoderm fate in the early mouse embryo.. Dev Biol.

[14] Ralston A, Rossant J (2005). Genetic regulation of stem cell origins in the mouse embryo.. Clin Genet.

[15] Jedrusik A, Parfitt DE, Guo G, Skamagki M, Grabarek JB (2008). Role of *Cdx2* and cell polarity in cell allocation and specification of trophectoderm and inner cell mass in the mouse embryo.. Genes Dev.

[16] Nishioka N, ichi Inoue K, Adachi K, Kiyonari H, Ota M (2009). The Hippo Signaling Pathway Components Lats and Yap Pattern Tead4 Activity to Distinguish Mouse Trophectoderm from Inner Cell Mass.. Dev Cell.

[17] Plusa B, Piliszek A, Frankenberg S, Artus J, Hadjantonakis AK (2008). Distinct sequential cell behaviours direct primitive endoderm formation in the mouse blastocyst.. Development.

[18] Meilhac SM, Adams R, Morris S, Danckaert A, Le Garrec JF (2009). Active cell movements coupled to positional induction are involved in lineage segregation in the mouse blastocyst.. Dev Biol.

[19] Alarcón VB, Marikawa Y (2008). Spatial alignment of the mouse blastocyst axis across the first cleavage plane is caused by mechanical constraint rather than developmental bias among blastomeres.. Mol Reprod Dev.

[20] Dallon JC, Othmer HG (2004). How cellular movement determines the collective force generated by the Dictyostelium discoideum slug.. J Theor Biol.

[21] Bischoff M, Parfitt DE, Zernicka-Goetz M (2008). Formation of the embryonic-abembryonic axis of the mouse blastocyst: relationships between orientation of early cleavage divisions and pattern of symmetric/asymmetric divisions.. Development.

[22] Fujimori T, Kurotaki Y, Miyazaki J, Nabeshima Y (2003). Analysis of cell lineage in two- and four-cell mouse embryos.. Development.

[23] Gardner RL (1997). The early blastocyst is bilaterally symmetrical and its axis of symmetry is aligned with the animal-vegetal axis of the zygote in the mouse.. Development.

[24] Piotrowska K, Wianny F, Pedersen RA, Zernicka-Goetz M (2001). Blastomeres arising from the first cleavage division have distinguishable fates in normal mouse development.. Development.

[25] Honda H, Motosugi N, Nagai T, Tanemura M, Hiiragi T (2008). Computer simulation of emerging asymmetry in the mouse blastocyst.. Development.

[26] Kimber SJ, Surani MA, Barton SC (1982). Interactions of blastomeres suggest changes in cell surface adhesiveness during the formation of inner cell mass and trophectoderm in the preimplantation mouse embryo.. J Embryol Exp Morphol.

[27] Piotrowska-Nitsche K, Zernicka-Goetz M (2005). Spatial arrangement of individual 4-cell stage blastomeres and the order in which they are generated correlate with blastocyst pattern in the mouse embryo.. Mech of Dev.

[28] Piotrowska-Nitsche K, Perea-Gomez A, Haraguchi S, Zernicka-Goetz M (2005). Four-cell stage mouse blastomeres have different developmental properties.. Development.

[29] Chazaud C, Yamanaka Y, Pawson T, Rossant J (2006). Early lineage segregation between epiblast and primitive endoderm in mouse blastocysts through the Grb2-MAPK pathway.. Dev Cell.

[30] Palsson E (2008). A 3-d model used to explore how cell adhesion and stiffness affect cell sorting and movement in multicellular systems.. J Theor Biol.

[31] Eyiyurekli M, Lelkes PI, Breen DE (2007). A computational system for investigating chemotaxis-based cell aggregation.. Advances in Artificial Life.

[32] Graner F, Glazier JA (1992). Simulation of biological cell sorting using a two-dimensional extended potts model.. Phys Rev Lett.

[33] Foty RA, Steinberg MS (2004). Cadherin-mediated cell-cell adhesion and tissue segregation in relation to malignancy.. Int J Dev Biol.

