# Supplementary figures and images for: Simulating the Mammalian Blastocyst - Molecular and Mechanical Interactions Pattern the Embryo
Source: PLoS Comput Biol. 2011 May 5;7(5):e1001128. doi: 10.1371/journal.pcbi.1001128 (PMC3088645; doi:10.1371/journal.pcbi.1001128)

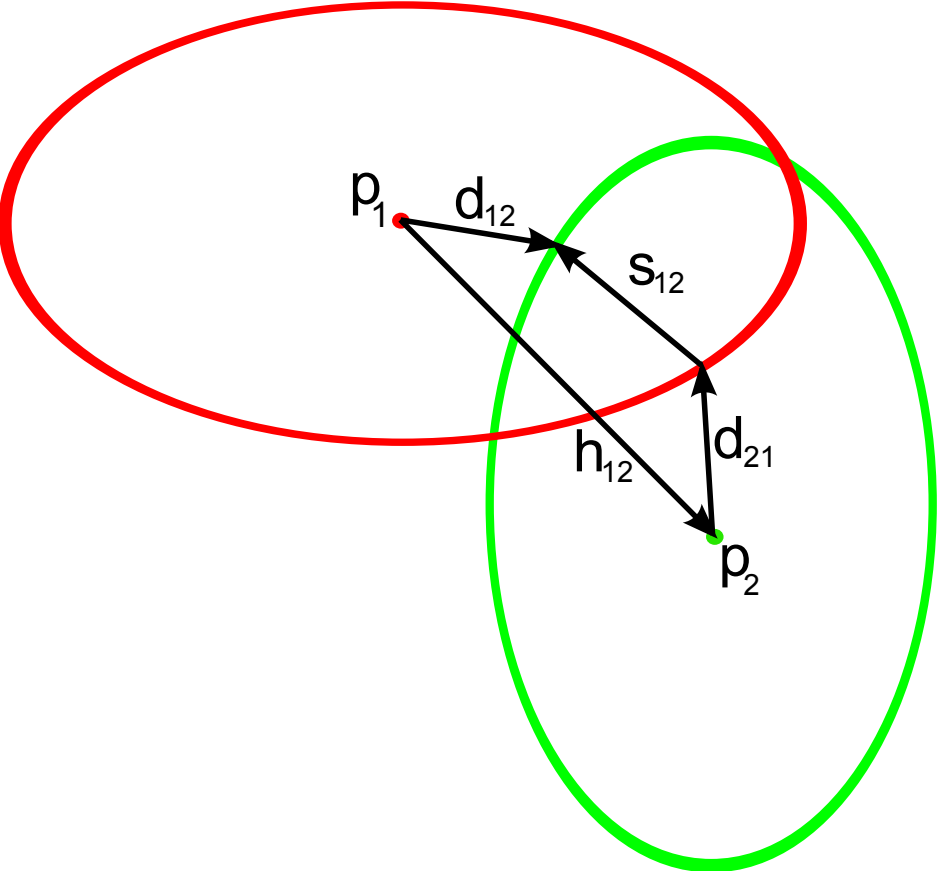

Supplement: Figure S1 — Schematic representation of the geometrical constructs used in force calculation of two interacting ellipsoidal cells. The geometry of each two cell intersection is approximately described in terms of vector between their centers , vectors between each cell center and a point on the ellipsoid closest to other ellipsoid's center , , and the vector between surfaces of the cells . The two cells are intersecting if . (0.01 MB PDF) [file pcbi.1001128.s001.pdf]

**CDX2 mRNA**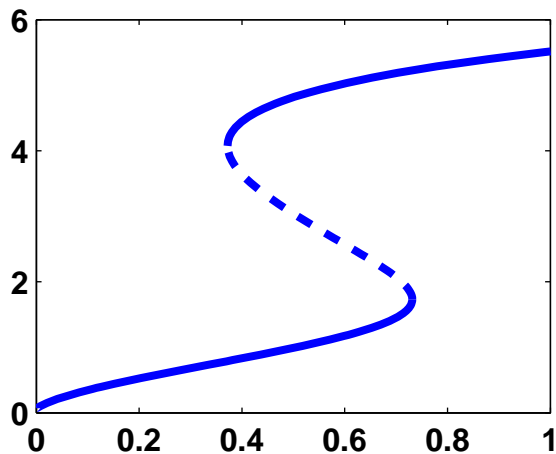**OCT4 mRNA**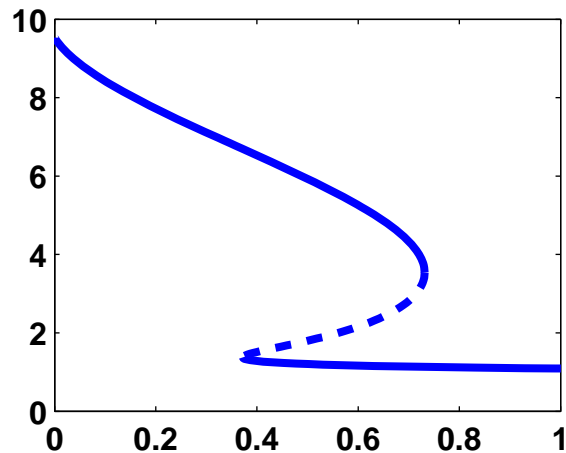**CDX2 Protein**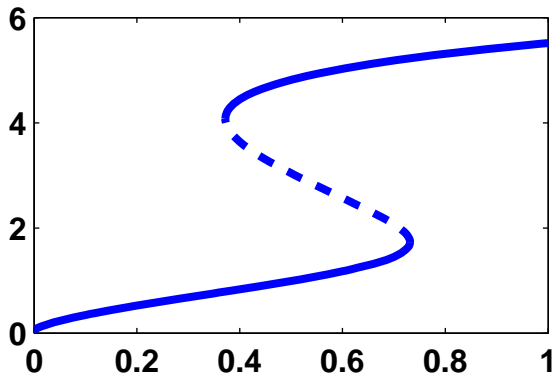**OCT4 Protein**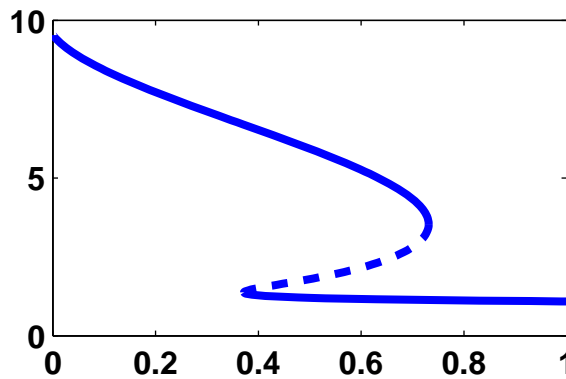

**Polarity signal P**

Supplement: Figure S2 — Bifurcation diagrams of both protein and mRNA equilibrium levels as a function of polarity signal P. Moderate values of P () result in bistable behavior of the system. (0.01 MB PDF) [file pcbi.1001128.s002.pdf]

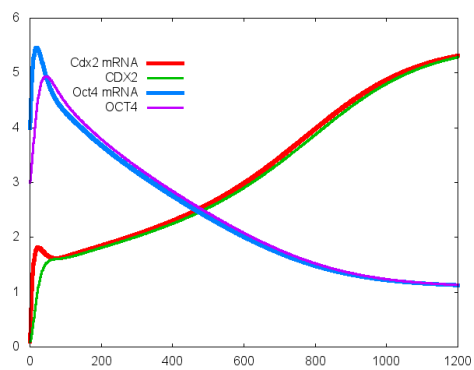

(a)

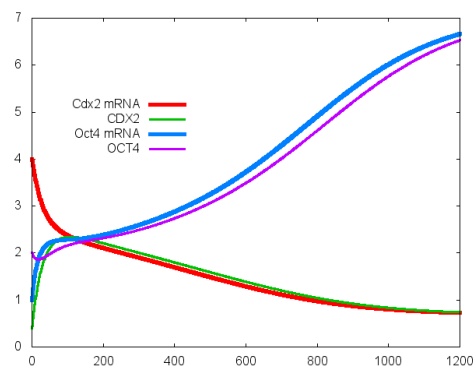

(b)

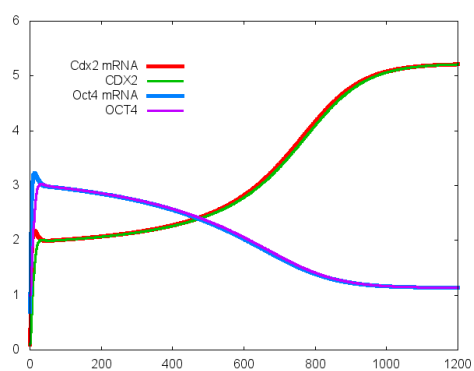

(c)

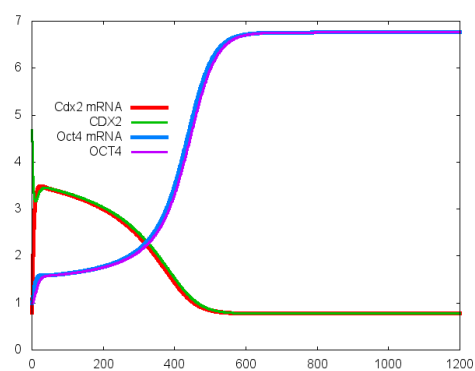

(d)

Supplement: Figure S3 — Time evolution of expression levels of Cdx2 and Oct4 showing switching from low to high CDX2 state and vice versa in position-based model network, (a) and (b), as well as in polarity based model (c) and (d). (0.04 MB PDF) [file pcbi.1001128.s003.pdf]

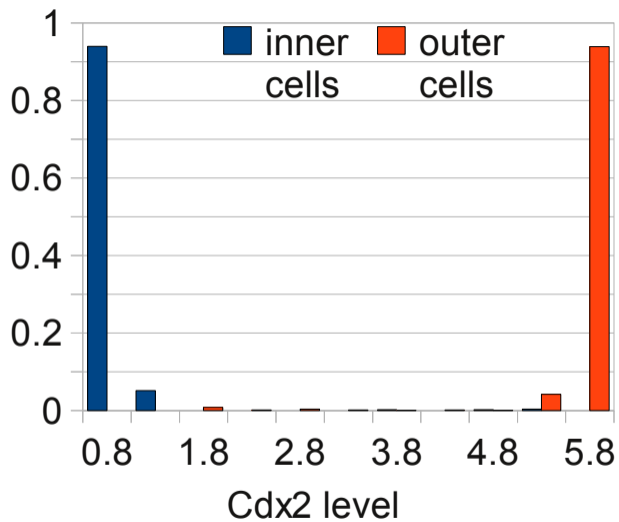

(a)

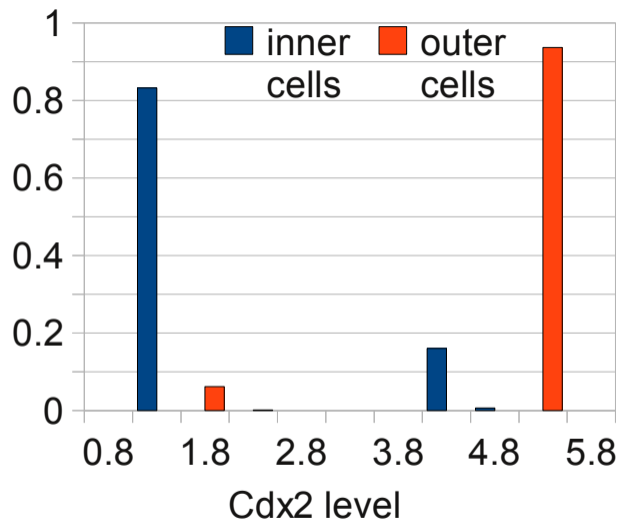

(b)

Supplement: Figure S4 — Histograms of CDX2 levels in the simulations of position-based (a) and polarity-based (b) models for the fast genetic network dynamics. Compare with Fig. 4 in the main text showing the case of the slow network. (0.04 MB PDF) [file pcbi.1001128.s004.pdf]

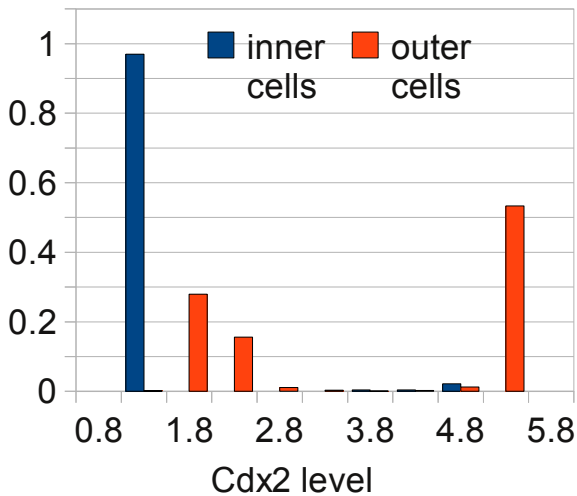

Supplement: Figure S5 — Aberrations of CDX2 levels in trophectoderm formation simulations in the case of non-optimized parameters in the polarity-based model. (0.02 MB PDF) [file pcbi.1001128.s005.pdf]

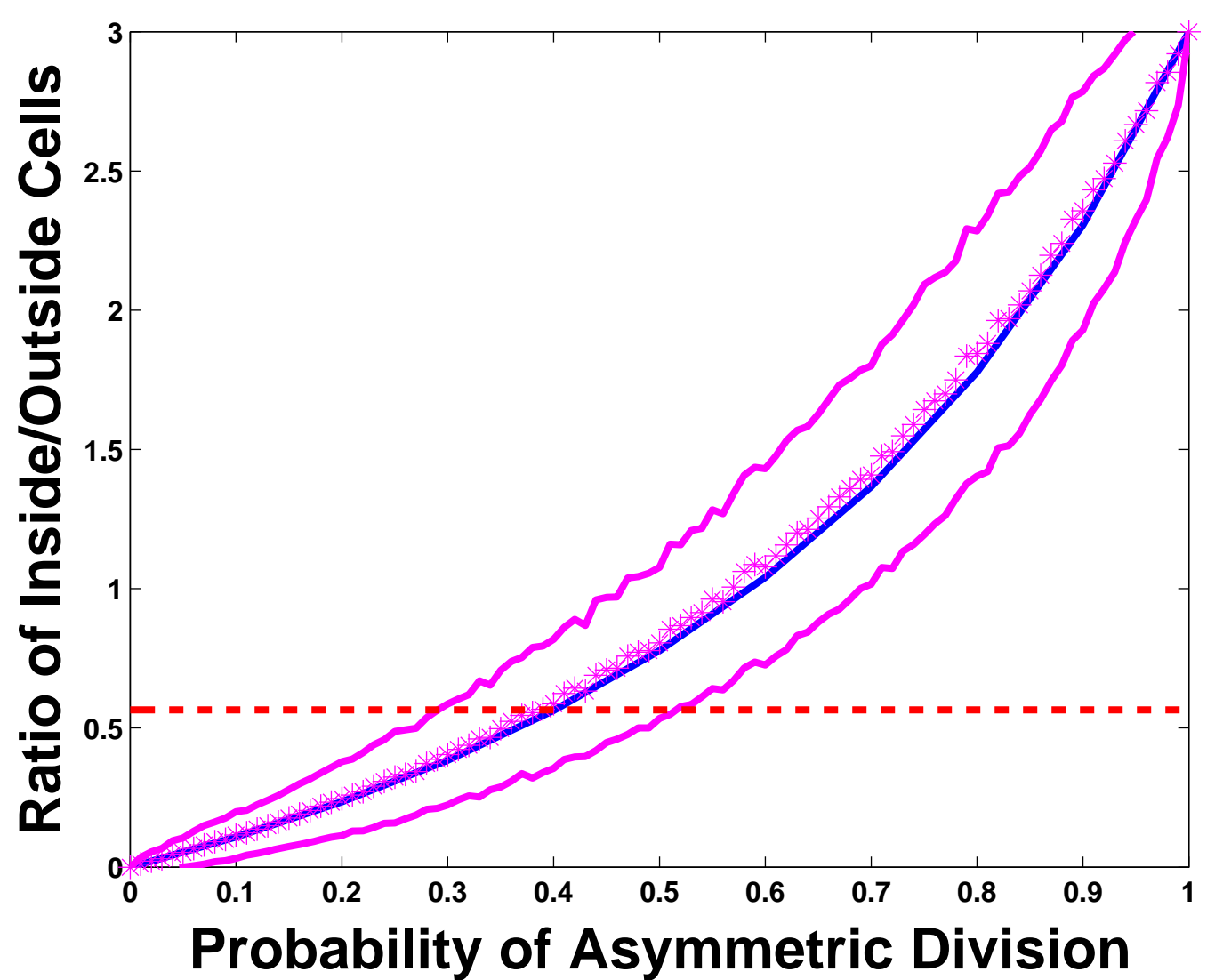

Supplement: Figure S6 — Results of the Monte Carlo analysis of the ratio of inner to outer cells at the 32-cell stage of embryo development as a function of the probability of asymmetric division. The theoretically estimated ratio of is marked by red horizontal dashed line. It crosses both the theoretical solution for corresponding asymmetric division probability (blue solid line) and the mean value of Monte Carlo simulations (pink stars) at . The pink solid lines mark the standard deviation of the Monte Carlo results. See Text S1 for details. (0.01 MB PDF) [file pcbi.1001128.s006.pdf]
