# Supplementary material for: Simulating the Mammalian Blastocyst - Molecular and Mechanical Interactions Pattern the Embryo
Source: PLoS Comput Biol. 2011 May 5;7(5):e1001128. doi: 10.1371/journal.pcbi.1001128 (PMC3088645; doi:10.1371/journal.pcbi.1001128)
Supplement: Table S1 — The ranges of the mechanical parameters used in the simulations. (0.03 MB PDF) [file pcbi.1001128.s007.pdf]

Supplementary table S1

zygote diameter - d [μm]	70 -130
cell elasticity - k [$nN/\mu m$]	5 - 20
cell adhesion - α [μN]	0 - 10
active forces - F_{act} [μN]	1 - 8
blastocoelic forces - F_{press} [μN]	1 - 5
random force frequency - f [1/cell cycle]	10 - 200
effective viscosity - μ [$\mu N \cdot s/\mu m$]	0.1 - 0.2

Table S1: The ranges of the mechanical parameters used in the simulations.
