# Supplementary material for: Simulating the Mammalian Blastocyst - Molecular and Mechanical Interactions Pattern the Embryo
Source: PLoS Comput Biol. 2011 May 5;7(5):e1001128. doi: 10.1371/journal.pcbi.1001128 (PMC3088645; doi:10.1371/journal.pcbi.1001128)
Supplement: Table S2 — Statistics of CDX2 expression in simulations of trophectoderm specification in cell polarity-based (gray columns) and position-based (white columns) models from 100 simulations. (0.02 MB PDF) [file pcbi.1001128.s008.pdf]

Supplementary table S2

cell type	sample size		average		median		1^{st}quartile		3^{rd}quartile	
	polar.	posit.	polar.	posit.	polar.	posit.	polar.	posit.	polar.	posit
inner cells	1004	948	1.03	0.87	0.8	0.6	0.8	0.57	0.8	0.79
outer cells	2196	2252	4.15	3.96	5.23	4.08	2.61	3.82	5.23	4.32

Table S2: Statistics of CDX2 expression in simulations of trophectoderm specification in cell polarity-based (gray columns) and position-based (white columns) models from 100 simulations.
