# Supplementary material for: Simulating the Mammalian Blastocyst - Molecular and Mechanical Interactions Pattern the Embryo
Source: PLoS Comput Biol. 2011 May 5;7(5):e1001128. doi: 10.1371/journal.pcbi.1001128 (PMC3088645; doi:10.1371/journal.pcbi.1001128)
Supplement: Table S3 — Parameters used in the simulations of the gene network in the position-based trophectoderm formation model. The parameters in the top table are the same as in polarity-based model. (0.03 MB PDF) [file pcbi.1001128.s009.pdf]

Supplementary table S3

F	a_0	a_1	a_2	a_3	b_0	b_1	b_2	n_1	n_2	T	γ_1	γ_2
10	0.01	0.25	1	1	0.5	0.25	1	2	1	20	1	20

P		τ
0.2	inner cell	150
1	outer cell	

Table S3: Parameters used in the simulations of the gene network in the position-based trophectoderm formation model. The parameters in the top table are the same as in polarity-based model.
