# Supplementary material for: Simulating the Mammalian Blastocyst - Molecular and Mechanical Interactions Pattern the Embryo
Source: PLoS Comput Biol. 2011 May 5;7(5):e1001128. doi: 10.1371/journal.pcbi.1001128 (PMC3088645; doi:10.1371/journal.pcbi.1001128)
Supplement: Table S4 — Parameters used in the simulations of the gene network in the polarity-based trophectoderm formation model. (0.03 MB PDF) [file pcbi.1001128.s010.pdf]

Supplementary table S4

F	a_0	a_1	a_2	a_3	b_0	b_1	b_2	n_1	n_2	T	γ_1	γ_2
10	0.01	0.25	1	1	0.5	0.25	1	2	1	20	1	20

P		τ
0.36	inner cell	30
0.73	outer cell	

Table S4: Parameters used in the simulations of the gene network in the polarity-based trophectoderm formation model.
