# Supplementary material for: Simulating the Mammalian Blastocyst - Molecular and Mechanical Interactions Pattern the Embryo
Source: PLoS Comput Biol. 2011 May 5;7(5):e1001128. doi: 10.1371/journal.pcbi.1001128 (PMC3088645; doi:10.1371/journal.pcbi.1001128)
Supplement: Text S1 — Supporting text. (0.14 MB PDF) [file pcbi.1001128.s011.pdf]

Supplementary Text S1

1 Model description

The models referred to in the main text, simulate the coupling of mechanical and molecular forces in the early stages of the development of mammalian embryo. The assumptions made in describing single cell mechanics follow the postulates introduced in [1, 2]. As in [1, 2], we describe the cell as an incompressible ellipsoid whose elastic response is characterized by three orthogonal springs placed along the three axes of the ellipsoid. In principle, deformations of the ellipsoid are allowed. However, in our simulations we find that, since the orientation of the ellipsoidal axes constrains available degrees of deformational freedom, arbitrary (non-dynamical) positioning of these axes leads to unphysical behavior or even instability of the solutions. Thus, even though the formalism presented here is applicable to ellipsoidal shapes we restrict ourselves in simulations to allowing for spherical shapes of the cells only.

1.1 Physical forces

Each cell (i) is subject to three types of forces from neighboring cells j; the elastic $F_{ij}^{elastic}$, adhesion $F_{ij}^{adhesion}$, and viscous drag F_{ij}^{drag} forces. Each of these forces depends upon the relative position of the cells and interaction parameters, which can differ between different cell types (for example we assume that cells expressing *Nanog* adhere together much more strongly than cells expressing *Gata6*). The geometry of cell overlap is shown in Figure S1.

To obtain the force on the cell due to the elastic deformation caused by other cell we assume that each cell deforms by $0.5\vec{s}_{ij}$ and calculate elastic response of each cell to such a stimulus. We obtain

$$F_{ij}^{elastic} = -f_e(\vec{s}_{ij}, i) + f_e(\vec{s}_{ji}, j). \quad (1)$$

where $f_e(\vec{s}, i)$ is the elastic response of the cell i to the compression by vector $0.5\vec{s}$, which when resolved to the ellipsoid axes is given by

$$f_e(\vec{s}, i) = g_a(\vec{s}, i)\hat{a}_i + g_b(\vec{s}, i)\hat{b}_i + g_c(\vec{s}, i)\hat{c}_i, \quad (2)$$

Here $\hat{a}_i$, $\hat{b}_i$ and $\hat{c}_i$ are the vectors of each of ellipsoid's axis and $g_a(\vec{s}, i) = f_{spring}(u_a, 0.5\vec{s} \cdot \hat{a}_i)$ is the response of each spring to the deformation. To model increased stiffness of the cell at larger deformations due to the presence of the organelles we choose nonlinear springs in the form

$$f_{spring}(u, \Delta u) = k(\Delta u + (\Delta u - 0.5u)^2 \Theta(\Delta u - 0.5u)), \quad (3)$$

where u is the length of undeformed spring and Θ a Heaviside step function.

The adhesion forces between cells contribute to the two effects. They cause the attractive normal forces and the drag forces. The attractive adhesion force acts on the cells that are separated by less than minimal interaction distance d_i^{adh}. That means that cells within the

adhesion range will be drawn closer to the point where elastic properties of the membranes will push them apart. This force is given by

$$F_{ij}^{adhesion} = \alpha_i A_{ij} \hat{s}_{ij} \text{ if } s_{ij} < d_i^{adh} \tag{4}$$

The coefficient α_i is the density of the adhesion molecules of cell i and A_{ij} is the area of intersection of adhesion interaction regions, which form ellipsoids extended by d_i^{adh} for each cell i. We approximate the area of intersection of these regions by the area of intersection of two spheres with radii $r_i = |\vec{d}_{ij} + 0.5\vec{s}_{ji}|$ and $r_j = |\vec{d}_{ji} + 0.5\vec{s}_{ij}|$,

$$A_{ij} = \pi \left[r_j^2 - \frac{1}{4} \left(h_{ij} - \frac{r_i^2 - r_j^2}{h_{ij}} \right)^2 \right], \tag{5}$$

where $\vec{h}_{ij}$ is a vector between the centers of both cells. Tangential drag force between cells i and j is given by

$$F_{ij}^{drag} = -\mu_{ij}(\vec{v}_i - \vec{v}_j)\frac{A_{ij}}{A_i}, \tag{6}$$

where $\vec{v}_i$ and $\vec{v}_j$ are velocities of cells i and j, μ_{ij} is the viscosity coefficient and A_{ij}/A_i is the ratio of the area of the cells' adhesion domains intersection to the total area of this region for cell i.

The forces acting on the cell are summed up over all the neighbors of the cell. The neighborhood relation itself is determined from the Voronoi diagram [3] of the cell centers, which is dynamically updated at each step of the simulation. The cells corresponding to adjacent Voronoi regions are then checked for actual overlap of interaction domains. We find that Voronoi relation is more appropriate as a description of neighborhood of a system of dividing and strongly interacting spherical cells than widely used proximity relation alone. The use of the latter in simulations with strong adhesion between cells could lead to non-physically large overlap of cells and the "collapse" of a cell cluster.

Since the movement of cells should be considered in a low Reynolds number regime, we assume that the acceleration of a cell can be neglected when simulating its dynamics, which leads to the equation of motion for the cell i

$$\dot{x}_i \sum_{j \in \mathcal{N}_i} \mu_{ij} \frac{A_{ij}}{A_i} = \sum_{j \in \mathcal{N}_i} F_{ij}^{elastic} + F_{ij}^{adhesion} + \mu_{ij} \frac{A_{ij}}{A_i} v_j, \tag{7}$$

which with the definitions

$$\begin{aligned} \mu_i &= \sum_{j \in \mathcal{N}_i} \frac{A_{ij}}{A_i} \mu_{ij}, \\ F_i^{elastic} &= \sum_{j \in \mathcal{N}_i} F_{ij}^{elastic}, \\ F_i^{adhesion} &= \sum_{j \in \mathcal{N}_i} F_{ij}^{adhesion}, \\ F_i^{drag} &= \sum_{j \in \mathcal{N}_i} \mu_{ij} \frac{A_{ij}}{A_i} v_j, \end{aligned}$$

becomes

$$\mu_i \dot{x}_i = F_i^{elastic} + F_i^{drag} + F_i^{adhesion} + \bar{F}_i, \tag{8}$$

The $\bar{F}_i$ above refers to additional external forces which we include in some simulations. For example, it may represent random forces simulating active movement of the cells or pressure forces from interaction with blastocoel.

In obtaining results displayed in Figure 2, the simulations from 1 to 32 cell stages were performed without any changes in adhesion and elasticity of the cells to emphasize the effects of geometrical and mechanical constraints. The graphs result from a single simulation. However, a similar behavior has been observed in all simulations. The size of the blastomers at the end of the simulation is determined by setting the size of the zygote to different values (from 70 to 130 micrometers) and performing five rounds of cleavages conserving the total cell volume. The size of the pellucid zone has been kept constant. In Figure 3, where we discuss the alignment of two cell embryo along the long axis of pellucid zone, this effect is very strong and can be observed in all simulations even with only slight elongation of the pellucid zone. The placement of the blastocoel to the one end of the same axis in our simulations is less regular and happens more frequently with larger elongation of pellucid zone. The expanding blastocoel was assumed to have no adhesion with the cells. Similarly, we estimated, from large movement of early embryo mass in experimental movies, that frictional forces between pellucid zone (ZP) and blastomers are negligible comparing to other forces. However, to check the effects of hypothetical forces opposing the motion of cells in contact with ZP we implemented those forces either as a drag force (proportional to tangential velocity of cells relative to ZP) or a frictional force (proportional to the normal force pressing the cell against ZP). If those frictional forces are present they slow down the movement of the embryo with respect to the ZP or cancel the movement completely, for moving forces smaller than friction. However, they do not change overall behavior of alignment of the embryo mass with respect to the ZP.

1.2 Cell division rules

During division, a cell of radius R with center at $\vec{x}$ is replaced by two cells of radii $r = R/\sqrt[3]{2}$ located at the points $\vec{x}_{1,2} = \vec{x} \pm (1 - \frac{1}{\sqrt[3]{2}})R\vec{n}$, where $\vec{n}$ is normal to the division plane. The direction of this normal is governed by the division rule associated with the cell type. Cells divide differently depending on (i) the expression of specific genes and (ii) their position within the embryo. Two division rules are used in our simulations:

- The *random division rule*, where the division plane direction is chosen randomly.
- The *polarized division rule*, where the division plane is either parallel or perpendicular to the vector normal to the pellucid zone surface.

Dividing cells, besides the obvious change of geometry, need to distribute their contents accordingly. Two types of rules which govern these distributions are:

- The *equal partition rule* divides cell contents symmetrically between two daughter cells. If data is an extensive property each cell receives half of the mother cell's amount. For intensive quantities, like concentrations of the proteins, both cells inherit the data of the mother cell.

- The *Asymmetric partition rule* distributes cell contents between daughter cells in unequal proportions given by the ratio $0 < r < 1$. Depending on their positions with respect to the division plane, one cell receives $D_1 = Dr$ while another gets $D_2 = D - D_1$ for extensive quantities, or $d_1 = 2rd$ and $d_2 = d - d_1$ respectively for intensive quantities.

1.3 Gene network dynamics

In addition, protein and mRNA levels in each cell evolve in time and are described by differential equations or other algebraic rules, corresponding to chemical reactions within the cell. Those reactions can differ for the different types of cells and can be turned on or off at specific times of the simulation. In this way practically any type of process can be simulated and spacial and temporal patterns of gene expression or protein distribution can be analyzed.

1.4 Implementation details

The model was implemented in the C++ programming language and visualizations of the output data were done with ParaView (Kitware) software [4]. The differential equations, for both the mechanics and the genetic networks, were solved with the 5-th order Runge-Kutta adaptive solver [5]. The mechanical and genetic solvers can be decoupled, in which case each is a separate instance of the Runge-Kutta solver with disjoint variables, or can be coupled and realized as a single instance of Runge-Kutta solver updating all the variables at the same time. The former method is useful when the time-scales of mechanics and the gene network are very different and the interactions between them are in one direction. The latter must be used in the case of convoluted interactions between mechanical and genetic cues. The simulations were performed an AMD Athlon(tm) 64 Processor 3500+ workstation. Single runs of embryonic development from 1 up to 256 cells required less than 15 minutes of wall clock time.

2 Simulations

Using the framework described above we performed simulations in order to elucidate the mechanisms behind important morphological events during pre-implantation embryogenesis. In a typical simulation scenario the cells start from the initial state (*e.g.* one zygote cell) and undergo a series of cleavages until the final condition (*e.g.* 64 cell count) is met. During the course of the simulation the mechanical and molecular cell data are modified according

to a set of predefined rules. We then analyze the results and compare it with experimental measurements of *e.g.* gene expression patterns and cell divisions.

2.1 Trophectoderm formation

In the simulation of trophectoderm formation we start from single oocyte cell of size 100 μm constrained by slightly larger and possibly elliptical pellucid zone. The simulation ends after five rounds of division take place (32 cell count) and the system is in mechanical equilibrium. The cells divide when their cell cycle length parameter exceeds the time from the last division. The first two cell cycles lengths are drawn from normal distribution with the mean of 1150 min and standard deviation of 150 min. The remaining three cell cycles are taken from normal distribution with the mean 750 min and standard deviation of 100 min.

One of the observations we aim to describe is how the cells, which form part of the trophectoderm, express *Cdx2* strongly. To this end, we simulate explicitly a modified network adapted from [6]. Furthermore, we describe two different hypotheses, as to how the trophectoderm layer of cells gets patterned, starting from a few cells [7][8][9]. The first hypothesis is "position-based", where the expression of *Cdx2* is dependent on where the cell is spatially located. The second is "polarity-based", where cells divide, depending on the concentration of CDX2. In both models we used the same genetic switch network based on the mutual repression between *Cdx2* and *Oct4* and auto-activation of both transcription factors [6]. Since the regulation of *Cdx2* mRNA is the crucial component of polarity-based model the network was extended to explicitly include mRNAs for both genes. We included also polarity signal P, enhancing *Cdx2* expression. Depending on the value of this signal, the network can have single, low or high CDX2 level state available, or it can show bistable behavior with respect to the levels of CDX2. The equations representing this network are,

$$\begin{aligned}
\tau_1 \frac{d[Crna]}{dt} &= \frac{F(a_0 + a_1[Cdx2]^{n_1} + a_3 P)}{1 + a_1[Cdx2]^{n_1} + a_2([Cdx2][Oct4])^{n_2} + a_3 P} - \gamma_1[Crna] \\
\tau_2 \frac{d[Cdx2]}{dt} &= T[Crna] - \gamma_2[Cdx2] \\
\tau_1 \frac{d[Orna]}{dt} &= \frac{F(b_0 + b_1[Oct4]^{n_1})}{1 + b_1[Oct4]^{n_1} + b_2([Cdx2][Oct4])^{n_2}} - \gamma_1[Orna] \\
\tau_2 \frac{d[Oct4]}{dt} &= T[Orna] - \gamma_2[Oct4]. \qquad (9)
\end{aligned}$$

In Figure S2 the steady state values of OCT4 and CDX2 are shown as functions of the polarity signal P for the parameters given in Table S3 and S4. We note that a bistable regime exists between the values of $P = 0.3720 - 0.7314$. Also shown in Figure S3 are the time series of the concentration of various molecules indicating that dependent upon the value of P, the polarity signal, which is different for the two models (see Table S3, S4 and Figure S3), the network can reach either switch ON(high CDX2), or switch OFF(low CDX2) state. At the 4-cell stage the concentration of CDX2 and OCT4 transcription factors in the blastomers is set to random and low values of $0.1 - 0.5$.

In each of the two cases we simulated both a "slow network", in which parameters describing biochemical interactions were chosen such that time of reaching chemical equilibrium within a cell was comparable with cell cycle length, and a "fast network" in which the time to equilibrate the network was much shorter than the length of the cell cycle. Both ways give consistent results in terms of robustness of the models in separating gene expression levels in inside and outside populations of cells. The "slow network" approach, however, displays much broader distributions thanks to the influence of stochastic effects of randomized initial conditions, cell division times and directions, see Figure 4. The "fast network" simulation, shown in Figure S4 is an extreme case where stochastic effects are completely overcome by the binary property of the switch, and the results clearly show the differences in the performance of the both models in the simulations. Since the details of the genetic networks are not a main focus of this work, the parameters for all networks were chosen by hand to obtain bistable behavior and no systematic optimization of parameters was performed.

2.1.1 Position-based model

In the position-based model it is assumed that cell lineage depends on the position of the cell in the cell cluster. Inner cells express *Oct4* and become inner cell mass (ICM) and outer cells express *Cdx2* and form the trophectoderm. We realized this behavior by setting the signal from polarity genes to the values which prevent bistable behavior of the network for both inner and outer cells. In such a case the outer cells have only high CDX2 state available for them and inner cells all tend to low CDX2 state. Inside or outside position of the cell in the embryo mass was decided by neighborhood relation based on Voronoi diagram of the cell centers. The parameters used in the implementation of position-based model are given in the Table S3. Blastomere cleavages take place in the random direction and are symmetric, *i.e.* the protein concentration is maintained in both daughter cells during division.

2.1.2 Polarity-based model

In the case of polarity-based model the dependence between gene expression and the position of the cell in the embryo is less direct than in the position-based model. The *Cdx2* mRNA is assumed to polarize to the apical side of the outer blastomers [8]. Therefore, during asymmetric cell division, it is partitioned such that the outer cell receives 90% and the inner cell 10% of the initial mRNA content. In addition, the probability of asymmetric division changes as

$$P_{asym} = \frac{c_{high} - [Cdx2]}{c_{high} - c_{low}}, \tag{10}$$

where c_{high} and c_{low} are high and low threshold levels of CDX2 concentration. In this way low CDX2 cells have larger chance to divide asymmetrically, than high CDX2 cells which prefer to divide symmetrically. Such set of rules results in the tendency of positioning high CDX2 cells in the outer layer of the embryo. Polarity signal in this case was set for inner and outer cells in the range which assured bistable behavior of network, so at each stage of simulations two states (low and high CDX2) are available for each cell. The parameters used for this network can be found in the Table S4. To achieve the best performance of this

model in proper distribution of CDX2 we fine tuned parameters of probability of asymmetric division, c_{high} and c_{low} with respect to the levels of CDX2 produced by network in Eqn. 9. In case such relation is not observed we obtain much worse performance of polarity-based model with large number of mispositioned low CDX2 cells, see Figure S5. For the results displayed in Figure 4, the statistics comes from 100 simulations of each model.

2.1.3 Consequence of asymmetric/symmetric cell divisions dependence on probabilities

In the previous section, the polarity rule assumed that the probabilities of symmetric/asymmetric divisions were dependent on the CDX2 level. Hence the ratio of inner to outer cells was dependent on the probabilities of a particular type of division. In this subsection, using a simple population model and with some rather simplified assumptions, we will analyze the dependence of the ratio of inner to outer cells as a function of the probability of asymmetric divisions. We will show that such a rule is **not very robust** in forming the required ratio. Let each cell be labeled with a polarity index P; polar cells $P = 1$ and apolar cells $P = 0$. An apolar cell divides to give two apolar daughter cells. A polar cell can either give one apolar and one polar cell, with probability p_0, or two polar cells with probability $p_1 = 1 - p_0$. In reality, these probabilities will depend on the concentration of CDX2 in some way, which will not concern us here. Starting from 8 cells we have two series of divisions to end up with 32 cells. Using these probabilities we can show that the final ratio of inner(apolar) to outer(polar) cells is,

$$\frac{p_0(4-p_0)}{(2-p_0)^2} \tag{11}$$

which is shown as a bold line in Figure S6. A Monte-Carlo simulation [1] of the same quantity is also shown in Figure S6. The simulation is obviously mirroring the analytic solution. The number of inner/outer cells can easily be read out by multiplying the ratio, for a given probability p_0 by 32. For example, at $p_0 \simeq 0.84$, $ratio \simeq 2$, which implies that $\simeq 21$ inner cells, $\simeq 11$ outer cells. Given this plot, it is evident that for higher p_0 the ratio of inner cells to outer cells keeps increasing. Let us now make some simple scaling arguments so as to approximate the number of inner to outer cells for the embryo.

Lets assume that each cell has volume v_0, which gives radius of $r = (\frac{3}{4\pi}v_0)^{1/3}$. If there are N_0 inner cells, then the total volume is $N_0 v_0$, which gives an equivalent surface area of $S = 4\pi(\frac{3}{4\pi}N_0 v_0)^{2/3}$. If we now assume that N_1 outer cells are placed on this surface area such that they completely cover all the N_0 inner cells. Each outer cell (same volume as inner cell) is pushed into the inner cell mass, such that it is 'half way' into the mass, *i.e.* it covers an area πr^2, then we need to satisfy,

$$N_1 \pi r^2 = S \tag{12}$$

[1]The MC simulation was done for a range of p_0, for each value of p_0, it was repeated 1000 times, so as to compute a mean and standard deviation. The analytic solution was obtained as follows: from 8–16 cells, assuming an initial template of $N = 8$, the number of $P = 1$ cells $N_{P1} = (2p_1 + p_0)N$, the number of $P = 0$ cells $N_{P0} = p_0 N$. Now for the next round of divisions from 16–32, we get the number of $P = 1$ cells $N_{P1} = (2p_1 + p_0)^2 N$, the number of $P = 0$ cells $N_{P0} = (2p_0 + 2p_0 p_1 + p_0{}^2)N$. Hence the ratio is as in Eq. 11

which after canceling all common factors leads to the following equation for N_0,

$$(32 - N_0)^3 = (8N_0)^2, \tag{13}$$

Using a nonlinear solver, we obtain $N_0 \simeq 11$, which implies a ratio of $\simeq 0.5652$. In the Figure 6 we indicate that for this to occur the probability of asymmetric division should be around $p_0 = 0.4$. Hence, p_0 must be controlled fairly tightly with $p_0 = 40\% \pm \delta$, in order to be able to faithfully form a covering of outer cells to the inner cell mass. Hence, this simple analysis suggests that to obtain the correct ratio of inside to outside cells, the genetic factors must exhibit tight control over the probability of symmetric/asymmetric divisions.

2.2 Endoderm formation

In endoderm formation simulation we concern ourselves with a situation where the blastocoel cavity is already developed and ICM cells express *Nanog* and *Gata6* in a "salt and pepper" pattern. We treat *Gata6* and *Nanog* expression as a binary, "on-off" state and assume that these both types of cells have different adhesion properties as described in the main text. The initial state of the simulation is 10 to 14 ICM cells (the number of cells in this range is chosen at random), randomly determined to be either *Nanog* or *Gata6* type. Starting from this condition we perform simulations of different cell separation mechanisms, including differential adhesion and oriented or random active movement of cells, and compare the results. Since the randomness in initial conditions and later division patterns can affect the outcome of the simulation we average the results over several runs.

2.2.1 Interaction with blastocoel

In case of the simulations, which treat blastocoelic surface as a static barrier for the ICM cells, we implement blastocoel as an impenetrable boundary limiting space available for the cells. In the simulations considering the dynamic aspects of the interaction of cells with blastocoel we model it by the pressure force (proportional to cross sectional area of cells) acting on the cells in contact with blastocoel in direction normal to ICM surface (approximately calculated from the neighbors positions).

2.2.2 Active movement forces

We include forces arising from the active movement of the cells, which can either be directional (with constant value and direction) towards blastocoel, or act in random direction due to enviromental noise. The magnitude and frequency of the random forces are chosen from the normal distributions centered at specified mean values. For the typical values of the forces and other mechanical parameters used in the simulations see Table S1.

References

[1] Dallon JC and Othmer HG. (2004) How cellular movement determines the collective force generated by the Dictyostelium discoideum slug. *J. Theor. Biol.* . 231(2):203-22.

[2] Palsson E and Othmer HG. (2000) A model for individual and collective cell movement in Dictyostelium discoideum. *Proc Natl Acad Sci U S A* 97(19):10448-53.

[3] Okabe A, Boots B and Sugihara K. Spatial Tessellations: Concepts and Applications of Voronoi Diagrams, 2nd ed. New York: Wiley, 2000.

[4] www.paraview.org

[5] Numerical Recipes: The Art of Scientific Computing, Third Edition (2007). *Cambridge University Press*

[6] Chickarmane V, Peterson C (2008) A computational model for understanding stem cell, trophectoderm and endoderm lineage determination. PLoS One 3: e3478.

[7] Tarkowski AK and Wroblewska J. (1967) Development of blastomers of mouse eggs isolated at the 4- and 8-cell stage.*J. Embryol. Exp. Morphol.* 18:155-80.

[8] Jedrusik A, Parfitt DE, Guo G, Skamagki M, Grabarek JB, Johnson MH, Robson P, Zernicka-Goetz M. (2008) Role of Cdx2 and cell polarity in cell allocation and specification of trophectoderm and inner cell mass in the mouse embryo.*Genes Dev.* 22:2692-706.

[9] Rossant J and Tam PPL. (2009) Blastocyst lineage formation, early embryonic asymmetries and axis patterning in the mouse.*Dev.* 136:701-13.
